# Supplementary material for: Correction of VWF multimerization in type 2A/IIC von Willebrand disease by exogenous VWF propeptide supplementation
Source: Mol Ther. 2025 Sep 16;33(12):6366–78. doi: 10.1016/j.ymthe.2025.09.027 (PMC12703184; doi:10.1016/j.ymthe.2025.09.027)
Supplement: Document S1. Figures S1–S5 and Tables S2 and S4 [file mmc1.pdf]

YMTHE, Volume *33*

Supplemental Information

Correction of VWF multimerization in type 2A/IIC von Willebrand disease by exogenous VWF propeptide supplementation

Ziqi Zhang, Qian Liang, Xiaoqian Xu, Yang Li, Changming Chen, Qiulan Ding, Aiwu Zhou, Wenman Wu, Xuefeng Wang, and Jing Dai

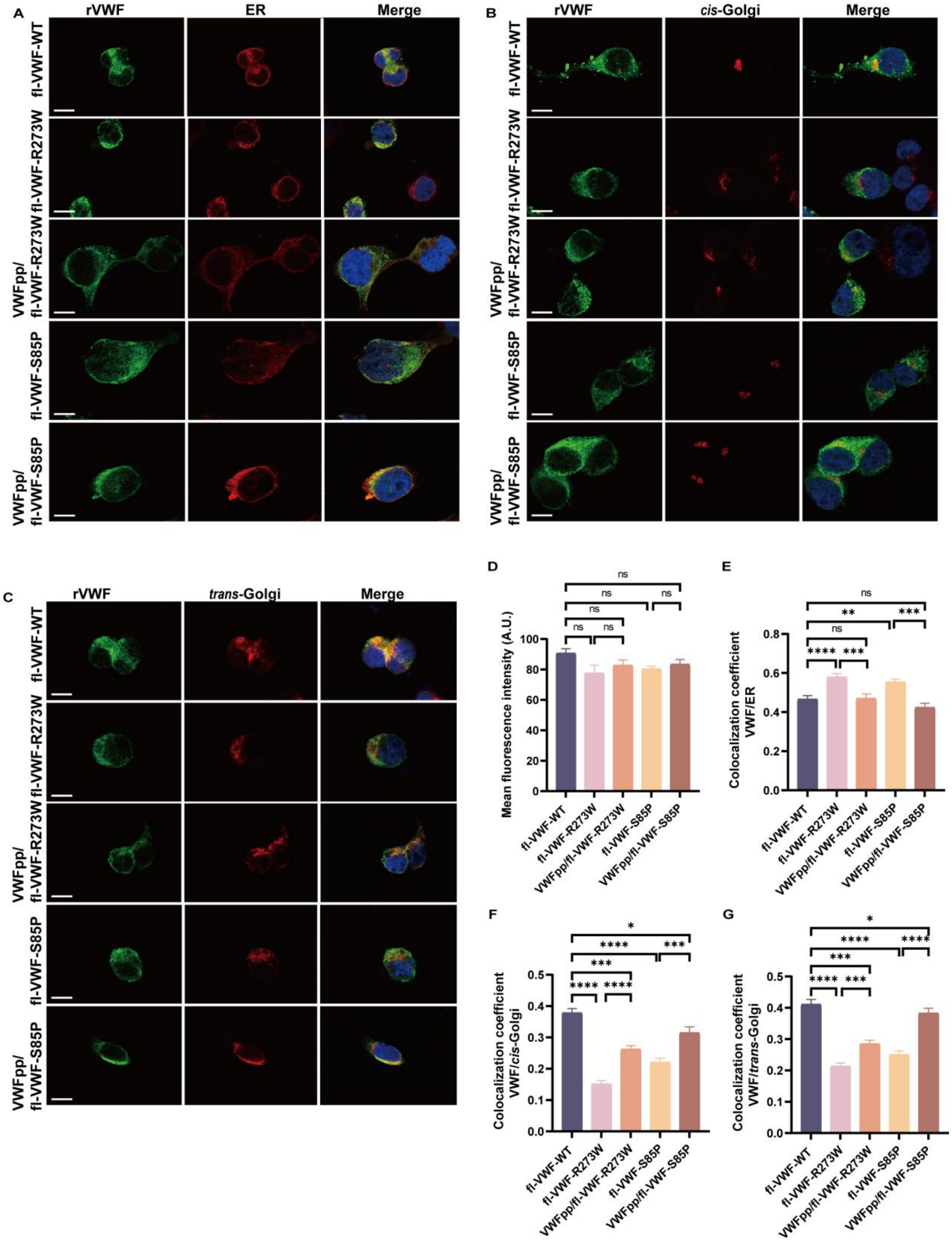
A
rVWF
ER
Merge
fl-VWF-WT
fl-VWF-R273W
VWFpp/
fl-VWF-R273W
fl-VWF-S85P
VWFpp/
fl-VWF-S85P
B
rVWF
cis-Golgi
Merge
C
rVWF
trans-Golgi
Merge
D
Mean fluorescence intensity (A.U.)
ns
E
Colocalization coefficient
VWF/ER
ns
**
***
****
F
Colocalization coefficient
VWF/cis-Golgi
*
****
***
G
Colocalization coefficient
VWF/trans-Golgi
fl-VWF-WT
fl-VWF-R273W
VWFpp/fl-VWF-R273W
fl-VWF-S85P
VWFpp/fl-VWF-S85P

Figure S1. Co-transfection with a plasmid expressing wild-type VWFpp improves intracellular trafficking of the type 3 VWD-causing variants.

(A-C) Representative images of AtT-20 cells transiently transfected with wild-type full-length VWF (fl-VWF-WT) or expression vectors for type 3 VWD-causing variants (fl-VWF-R273W and fl-VWF-S85P), or co-transfected with VWFpp and fl-VWF at a 2:1 molar ratio, were stained for VWF (green), the endoplasmic reticulum (ER) (red), the *cis*-Golgi (red), and the *trans*-Golgi (red). Nuclei were stained with 4’,6-diamidino-2-phenylindole (DAPI, blue). Scale bar = 10 μm. (D) The mean fluorescence intensity of intracellular rVWF-WT, rVWF-R273W, and rVWF-S85P mutants, with and without co-transfection of wild-type VWFpp, was measured in arbitrary units (A.U.) for at least 50 positively transfected cells from two independent experiments. (E-G) The degree of co-localization of different species of rVWF with the ER, *cis*-Golgi and *trans*-Golgi. Error bars represent the standard error of mean (SEM). *, $P < 0.05$; **, $P < 0.01$; ***, $P < 0.001$; ****, $P < 0.0001$; ns, not statistically significant.

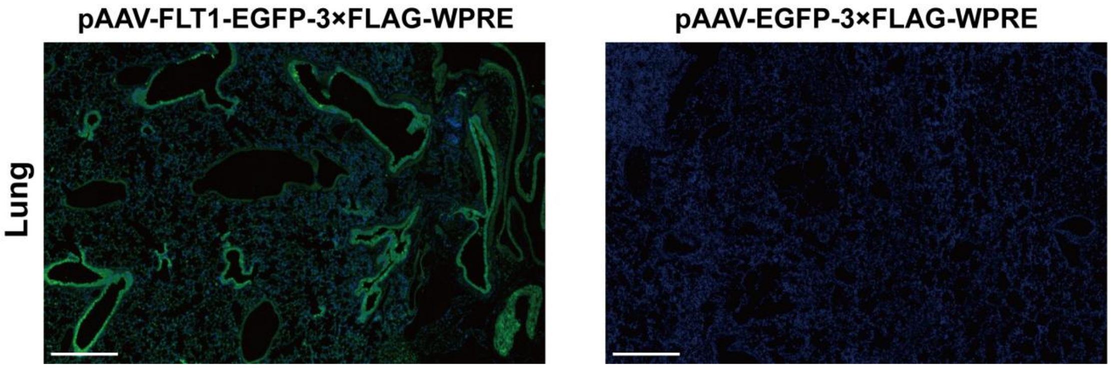

Figure S2. Representative GFP immunofluorescence staining of mouse lung tissue. The left panel shows a mouse injected with the AAV expression vector for mVWFpp under the control of the endothelial-specific promoter fms-like tyrosine kinase 1 (FLT1). The right panel shows a mouse injected with the control AAV vector lacking the FLT1 promoter. Green fluorescence indicates GFP expression, and blue fluorescence indicates DAPI nuclear staining. Scale bar: 500 μm.

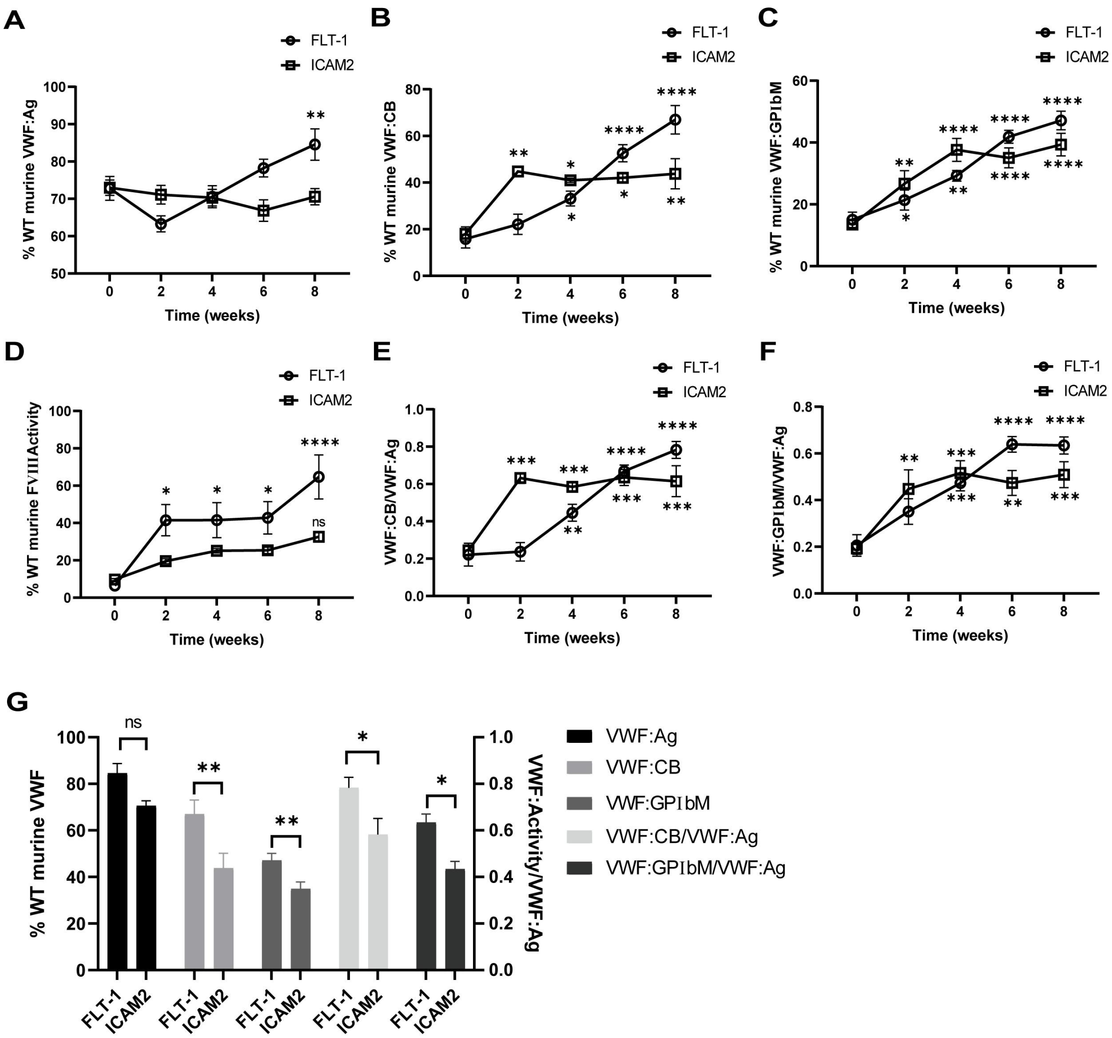

Figure S3. Restoration of VWF activity in a type 2A VWD mouse model via AAV-mediated delivery of wild-type murine VWF propeptide under the control of two distinct endothelial-specific promoters. (A-F) VWF antigen levels (VWF:Ag), VWF collagen binding activity levels (VWF:CB), VWF GPIb-binding activity levels (VWF:GPIbM), FVIII activity levels and the ratios of VWF:CB/VWF:Ag and VWF:GPIbM/VWF:Ag in type 2A VWD mouse plasma ($VWF^{Y87S/Y87S}$) before and after tail vein administration of AAV vectors delivering wild-type VWFpp at a dose of 5.0×10^{11} vg per mouse. Gene expression was driven by either the endothelial-specific fms-like receptor tyrosine kinase 1 (FLT1) or intercellular adhesion molecule 2 (ICAM2) promoter (n = 8). (G) Comparison of VWF:Ag levels, VWF:CB levels, VWF:GPIbM levels, VWF:CB/VWF:Ag ratios and VWF:GPIbM/VWF:Ag ratios between the two promoter groups 8 weeks post-gene transfer. Data are presented as mean ± standard error of the mean (SEM). *, P < 0.05; **, P < 0.01; ***, P < 0.001; ****, P < 0.0001; ns, not statistically significant.

A

Cell lines	rVWF:Ag	rVWF:CB	rVWF:GPIbM	rVWF:CB/rVWF:Ag	rVWF:GPIbM/rVWF:Ag
AtT20	100.0 ± 0.0	100.0 ± 0.0	100.0 ± 0.0	1.0 ± 0.0	1.0 ± 0.0
HEK293	83.8 ± 5.7	75.0 ± 6.4	82.4 ± 7.3	0.9 ± 0.0	0.9 ± 0.1
CHO	75.2 ± 7.2	67.5 ±10.1	57.1 ± 6.6	0.9 ± 0.1	0.8 ± 0.0

B

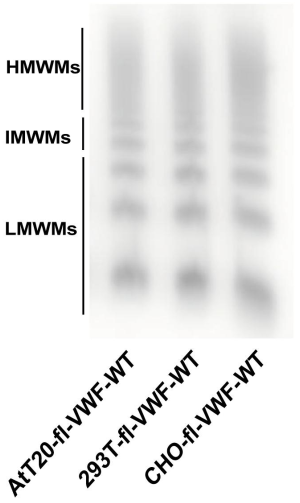

C

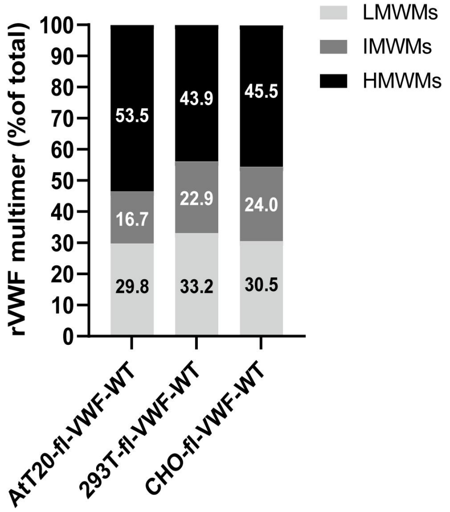

Figure S4. Expression of full-length wild-type recombinant VWF in AtT-20, HEK293, and CHO cells. (A) *In vitro* expression of full-length wild-type recombinant VWF performed in three different cell lines. VWF expression levels in HEK293, and CHO cells were calculated as a percentage of those expressed in AtT20 cells. (B) Representative image of the VWF multimer pattern in the conditioned medium of AtT-20 cells, HEK 293 cells and CHO cells transiently expressing full-length wild-type VWF. HMWMs: high-molecular-weight VWF multimers (bands >5); IMWMs: intermediate-molecular-weight VWF multimers (bands 4-5); LMWMs: low-molecular-weight VWF multimers (bands 1-3). (C) Relative quantification of HMWMs, IMWMs, and LMWMs from panel B, based on band intensity measured using Image J software.

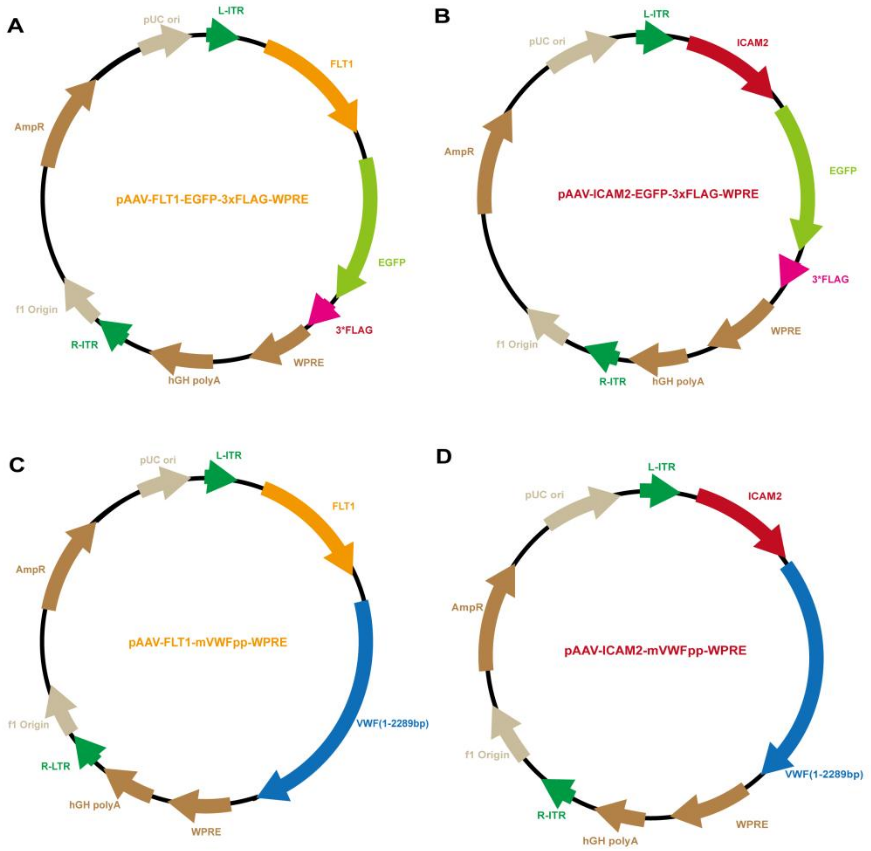

Figure S5. Maps of AAV vector plasmids.

(A-B) Schematic representation of control AAV vector plasmids utilizing fms-like receptor tyrosine kinase 1 (FLT1) or intercellular adhesion molecule 2 (ICAM2) as endothelial-specific promoters. The control vectors also contain additional elements, including enhanced green fluorescent protein (EGFP) as a reporter, a triple FLAG peptide (3×FLAG), and the woodchuck hepatitis virus posttranscriptional regulatory element (WPRE). (C-D) Schematic representation of test AAV vector plasmids expressing murine VWF propeptide (VWFpp), generated by replacing the EGFP and triple FLAG peptide coding regions with the wild-type murine VWFpp sequence.

Table S1. *In vitro* restoration of VWF multimerization and secretion by co-expression of wild-type VWF propeptide with type 2A VWD variants at different molar ratios.

VWFpp, VWF propetide; fl-hVWF, full length human VWF;[a], the stepwise assembly of VWF multimer guided by VWFpp. Step i, formation of VWFpp dimers through D2:D2 interactions; step ii, recruitment of the D'D3 domain; step iii, helical stacking of intertwined D1D2D'D3 dimers via D1:D2 interactions. [b], The levels of rVWF:Ag, rVWF:CB, rVWF:GPIbM, the rVWF:CB/rVWF:Ag ratios and rVWF:GPIbM/rVWF:Ag ratios in the conditioned medium transfected with mutant fl-hVWF plasmids alone. [c], The levels of rVWF:Ag, rVWF:CB, rVWF:GPIbM, the rVWF:CB/rVWF:Ag ratios and rVWF:GPIbM/rVWF:Ag ratios observed after co-transfection of mutant fl-hVWF with hVWFpp plasmids at various molar ratios. The molar ratios of hVWFpp to mutant fl-hVWF plasmids that achieved the best correction effect are highlighted in bold. NA, not applicable.

Table S2. VWF phenotypic characterization in wild-type and type 2A/IIC mice (VWF$^{Y87S/Y87S}$).

Genotype	VWF:Ag	VWF:CB	VWF:CB/VWF:Ag
VWF$^{WT/WT}$(wild-type)	84.8±15.8	80.2±21.8	0.9±0.2
VWF$^{Y87S/Y87S}$(homozygotes)	68.9±8.5	15.8±10.2	0.2±0.1
VWF$^{Y87S/WT}$(heterozygotes)	68.1±10.8	41.9±17.9	0.6±0.2

Table S3. Complete blood count analysis in the type 2A/IIC VWD mouse model (VWF$^{Y87S/Y87S}$) 16 weeks post vector administration.

WBC, White Blood Cell Count (Reference range: 4.45-13.96×10^9/L); NEU(%), Neutrophil Percentage (Reference range: 7.36-28.59%); LYM(%), Lymphocyte Percentage (Reference range: 61.26-87.18%); MON(%), Monocyte Percentage (Reference range: 2.18-11.02%); EOS(%), Eosinophil Percentage (Reference range: 0.13-4.42%); BAS(%), Basophil Percentage (Reference range: 0.01-1.24%); NEU(10^9/L), Neutrophil Count (Reference range: 0.53-3.09×10^9/L); LYM(10^9/L), Lymphocyte Count (Reference range: 3.24-11.15×10^9/L); MON(10^9/L), Monocyte Count (Reference range: 0.15-0.94×10^9/L); EOS(10^9/L), Eosinophil Count (Reference range: 0.01-0.42×10^9/L); BAS(10^9/L), Basophil Count (Reference range: 0-0.13×10^9/L); RBC, Red Blood Cell Count (Reference range: 7.14-12.2×10^{12}/L); HGB, Hemoglobin Concentration (Reference range: 108.0-192.0 g/L); HCT, Hematocrit (Reference range: 37.3-.0%); MCV, Mean Corpuscular Volume (Reference range: 42.7-56.0 fL); MCH, Mean Corpuscular Hemoglobin (Reference range: 11.7-16.3 pg); MCHC, Mean Corpuscular Hemoglobin Concentration (Reference range: 246.0-349.0 g/L); RDW-SD, Red Blood Cell Distribution Width- Standard Deviation (Reference range: 23.0-39.0 fL); RDW-CV, Red Blood Cell Distribution Width-Coefficient of Variation (Reference range: 12.0-19.0%); PL, Platelet Count (Reference range: 841-2159×10^9/L); MPV, Mean Platelet Volume (Reference range: 4.3-6.1 fL); PCT, Platelet Hematocrit (Reference range: 0.1-0.78%); PDW, Platelet Distribution Width (Reference range: 12.0-17.5%).

Table S4. *In vitro* restoration of VWF multimerization by co-expression wild-type VWF propetptide for p.Tyr87Ser variant in three cell lines.

Cell lines	rVWF:Ag (% of WT)		rVWF:CB (% of WT)		rVWF:GPIbM(% of WT)		rVWF:CB/rVWF:Ag		rVWF:GPIbM/rVWF:Ag	
	Pre[a]	Post[b]	Pre[a]	Post[b]	Pre[a]	Post[b]	Pre[a]	Post[b]	Pre[a]	Post[b]
AtT20	111.1 ± 11.8	90.9 ± 11.3	8.2 ± 2.2	37.3 ± 7.3	28.1 ± 6.4	60.6 ± 9.5	0.1 ± 0.0	0.4 ± 0.2	0.2 ± 0.1	0.6 ± 0.1
HEK293	87.1 ± 8.4	69.6 ± 7.0	5.4 ± 4.2	15.4 ± 2.3	21.8 ± 6.3	30.9 ± 8.1	0.1 ± 0.1	0.2 ± 0.1	0.2 ± 0.2	0.4 ± 0.2
CHO	81.4 ± 9.2	57.1 ± 7.7	2.1 ± 1.3	12.0 ± 2.5	26.4 ± 9.1	27.4 ± 9.1	0.1 ± 0.0	0.2 ± 0.1	0.2 ± 0.2	0.4 ± 0.1

[a], The levels of rVWF:Ag, rVWF:CB, and the rVWF:CB/rVWF:Ag ratios in the conditioned medium transfected with mutant full length VWF plasmids alone. [b], The levels of rVWF:Ag, rVWF:CB, and the rVWF:CB/rVWF:Ag ratios observed after co-transfection of mutant full length VWF with human VWFpp plasmids at a 4:1 molar ratio.
