# Supplementary material for: Correction of VWF multimerization in type 2A/IIC von Willebrand disease by exogenous VWF propeptide supplementation
Source: Mol Ther. 2025 Sep 16;33(12):6366–78. doi: 10.1016/j.ymthe.2025.09.027 (PMC12703184; doi:10.1016/j.ymthe.2025.09.027)
Supplement: Document S2. Article plus supplemental information [file mmc4.pdf]

Molecular Therapy
Original Article

Correction of VWF multimerization in type 2A/IIC von Willebrand disease by exogenous VWF propeptide supplementation

Ziqi Zhang,[1,2,7] Qian Liang,[1,2,7] Xiaoqian Xu,[2,3,4,7] Yang Li,[1,2] Changming Chen,[1,2] Qiulan Ding,[1,2,5] Aiwu Zhou,[6] Wenman Wu,[1,5] Xuefeng Wang,[1,5] and Jing Dai[1]

[1]Department of Laboratory Medicine, Ruijin Hospital, Shanghai Jiaotong University School of Medicine, Shanghai 200025, China; [2]State Key Laboratory of Medical Genomics, Shanghai Institute of Hematology, Ruijin Hospital, Shanghai Jiaotong University School of Medicine, Shanghai 200025, China; [3]Department of Hematology, Ruijin Hospital, Shanghai Jiao Tong University School of Medicine, Shanghai 200025, China; [4]National Research Center for Translational Medicine at Shanghai, Shanghai Institute of Hematology, Shanghai 200025, China; [5]Collaborative Innovation Center of Hematology, Shanghai Jiaotong University School of Medicine, Shanghai 200025, China; [6]Institute for Translational Medicine on Cell Fate and Disease, Shanghai Ninth People's Hospital, Key Laboratory of Cell Differentiation and Apoptosis of National Ministry of Education, Department of Pathophysiology, Shanghai Jiao Tong University School of Medicine, Shanghai 200023, China

Gene therapy remains the only cure for von Willebrand disease (VWD), but it is limited by the large von Willebrand factor (VWF) gene size. Variants affecting the VWF propeptide (VWFpp) impair multimerization, causing type 2A/IIC VWD. VWFpp serves as a pH-sensitive template for VWF multimer assembly, suggesting that in *trans* VWFpp supplementation may restore multimerization in VWF variants with defective propeptides. Co-expression of wild-type VWFpp with mutant full-length VWF *in vitro* led to modest yet consistent improvements in the VWF multimer profile across eight type 2A/IIC VWD-causing variants. Notably, variants with defect D2:D2 interface required lower levels of VWFpp for multimerization rescue, whereas those with intact D2:D2 interfaces exhibited a greater demand. Furthermore, a transgenic mouse model of type 2A/IIC VWD carrying the p. Tyr87Ser mutation was treated with an AAV9 vector encoding VWFpp under the control of endothelial-specific promoters. VWFpp administration remarkably restored VWF multimerization, increased VWF:CB levels from 15.8% ± 10.2% to 71.2% ± 12.7% for at least 16 weeks, corrected the bleeding tendency and improved platelet function. Both *in vitro* and *in vivo* findings demonstrate that in *trans* VWFpp supplementation can rectify defects in VWF multimerization caused by variants in VWFpp, offering a novel therapeutic strategy for type 2A/IIC VWD.

INTRODUCTION

von Willebrand disease (VWD) is the most common inherited bleeding disorder, resulting from either quantitative (types 1 and 3) or qualitative (type 2) deficiencies of von Willebrand factor (VWF), a multimeric plasma glycoprotein essential for platelet adhesion and aggregation at sites of vascular injury and for stabilizing coagulation factor VIII (FVIII) in circulation.[1] VWD patients with low VWF activity levels are at an increased risk of bleeding and often require long-term prophylaxis.[2] It is necessary to maintain VWF activity above 0.50 IU/mL to prevent bleeding during surgery and invasive procedures, and elevated VWF levels can help protect female VWD patients from excessive menstrual bleeding.[2] Desmopressin and plasma-derived VWF/FVIII concentrates are the primary therapeutic options for bleeding management in VWD patients.[3,4] However, due to the relatively short half-life of VWF, approximately 12 h, frequent dosing is often required to sustain hemostatic efficacy.[3–5]

Gene therapy remains the only curative approach for VWD; however, the large size of the *VWF* gene, which encodes an 8.4-kb mRNA transcript, presents a major challenge for delivery via viral vectors, particularly the commonly used adeno-associated virus (AAV) vectors. Efforts to circumvent the AAV packaging limitations by splitting the VWF coding region between two AAV vectors or incorporating full-length VWF cDNA into lentiviral vectors have thus far failed to achieve efficient *in vivo* VWF expression.[6–8] Furthermore, gene therapy strategies based on full-length VWF expression may be further hindered by the presence of dominant-negative VWF variants in host cells. To overcome these limitations, alternative approaches have been explored. Rather than attempting to express full-length VWF, one study sought to partially restore the FVIII-stabilizing function of

Received 12 April 2025; accepted 12 September 2025;
https://doi.org/10.1016/j.ymthe.2025.09.027.

[7]These authors contributed equally

Correspondence: Wenman Wu, Department of Laboratory Medicine, Ruijin Hospital, Shanghai Jiaotong University School of Medicine, Shanghai 200025, China.
E-mail: wenmanwu@yahoo.com
Correspondence: Xuefeng Wang, Department of Laboratory Medicine, Ruijin Hospital, Shanghai Jiaotong University School of Medicine, Shanghai 200025, China.
E-mail: wxf63@shsmu.edu.cn
Correspondence: Jing Dai, Department of Laboratory Medicine, Ruijin Hospital, Shanghai Jiaotong University School of Medicine, Shanghai 200025, China.
E-mail: dj40572@rjh.com.cn

 © 2025 The Authors.
Published by Elsevier Inc. on behalf of The American Society of Gene and Cell Therapy.
This is an open access article under the CC BY license (http://creativecommons.org/licenses/by/4.0/).

VWF by expressing the N-terminal portion of VWF (D1-D2-D′-D3 domains).[9] Another approach utilized small interfering RNA to selectively silence the expression of endogenous dominant-negative VWF mutants, thereby mitigating their detrimental effects.[9,10]

Type 2A VWD is characterized by a selective deficiency of high-molecular-weight multimers (HMWMs), which are essential for the VWF to perform its function in hemostasis. Impaired multimer assembly caused by variants in VWF propeptide (VWFpp) is historically designated “type 2A/IIC,” accounting for 4% of all type 2A VWD cases.[11] The recent elucidation of the structural basis of VWF multimerization provides novel insights into the development of gene therapy approaches for VWD caused by dysfunctional VWFpp. VWFpp is synthesized as part of the VWF precursor and assists in the proper folding of the VWF protein within the endoplasmic reticulum (ER). It also regulates VWF transporting from the ER to the Golgi apparatus. Variants in VWFpp can lead to the retention of VWF in the ER and reduced secretion, which is observed in some cases of type 1 and type 3 VWD. Once in the *trans*-Golgi network, VWFpp is cleaved from mature VWF by the proprotein convertase furin and acts as a pH-sensitive template for VWF multimerization.[12] The homodimer of VWFpp is first formed by interactions between D2 domains at acidic pH (step 1), and two D′D3 domains are later recruited to form an intertwined D1D2D′D3 homodimer (step 2), which then piles up through the D1:D2 interface (step 3) and forms VWF multimer through a disulfide bond between two D3 domains (step 4). Variants affecting the role of VWFpp in multimerization contribute to type 2A/IIC VWD, characterized by selective loss of HMWMs.[13] *In vitro* co-expression studies using plasmids encoding VWFpp and mature VWF have demonstrated that VWFpp can function in *trans* to direct the assembly of VWF multimers and the formation of Weibel-Palade bodies (WPBs),[14,15] indicating that covalent linkage between VWFpp and mature VWF is not a prerequisite for multimer formation. This distinctive cellular machinery for VWF multimerization presents a therapeutic opportunity to correct defective multimer assembly in type 2A/IIC VWD by delivering only wild-type (WT) VWFpp to compensate for the dysfunctional endogenous VWFpp.

In this study, we first examined the capacity of co-expressed WT human VWFpp to rescue the multimerization and secretion of VWF mutants affecting VWFpp in association with both type 2A/IIC and type 3 VWD *in vitro*. We then assessed the efficacy of AAV-mediated delivery of murine VWFpp (mVWFpp) in restoring VWF multimerization and ameliorating the bleeding phenotype in a transgenic mouse model of human type 2A/IIC VWD carrying the VWFpp p.Tyr87Ser mutation.

RESULTS

Co-transfection of VWFpp in AtT-20 cells partially restored VWF multimerization and secretion in VWF variants associated with type 2A/IIC VWD

The *in vitro* corrective effect of VWFpp in restoring VWF multimerization was assessed in eight type 2A/IIC VWD-associated variants. The eight variants were p.Asp437_Arg442del and p.Cys570Ser, which are predicted to disrupt VWFpp dimer formation (step 1); p.Ser292_Glu333delinsLys, p.Cys379Gly, and p.Gly550Arg, which likely impair D′D3 recruitment (step 2); and p.Tyr87Ser, p.Asp172Asn, and p.Arg202Trp, which are predicted to interfere with helical stacking (step 3). The recombinant VWF:antigen (rVWF:Ag) and rVWF:collagen binding (CB) levels in the conditioned medium from cells transfected with WT full-length VWF (fl-VWF) were approximately 13.4% and 11.8%, respectively, of those in normal pooled plasma. In the conditioned medium transfected with mutant fl-VWF alone, the absence of HMWMs and a significantly reduced rVWF:CB/rVWF:Ag (0.1 ± 0.1) and rVWF:GP-binding capacity (GPIbM)/rVWF:Ag ratio (0.1 ± 0.1) were observed, reflecting the characteristic laboratory phenotype of type 2A/IIC VWD (Figure 1; Table 1). Co-transfection of VWFpp increased rVWF:Ag levels to >50% and significantly improved the VWF multimer profile in a dose-dependent manner across all variants (Figure 1; Tables 1 and S1), increased rVWF:CB levels to 20%–40% of WT, and simultaneously enhanced the GPIb binding capacity. Notably, for variants with disruptions in the D2:D2 interface (p.Asp437_Arg442del and p.Cys570Ser), the maximal rescue of multimerization was achieved at a 1:1 molar ratio of VWFpp to mutant fl-VWF plasmid. In contrast, for variants with intact D2:D2 interfaces (those affecting steps 2 and 3), a larger amount of external VWFpp was required to block the defective VWFpp, and optimal multimerization rescue was observed at a 2:1 or 4:1 molar ratio (Figure 2; Tables 1 and S1).

Co-transfection of VWFpp in AtT-20 cells resulted in a modest increase in rVWF:Ag levels for VWF variants associated with type 3 VWD

The *in vitro* corrective effect of VWFpp on VWF secretion was evaluated in three type 3 VWD-associated variants. Transfection with mutant plasmids alone led to near-undetectable VWF levels in the conditioned medium, mirroring the type 3 VWD phenotype. Co-transfection with VWFpp resulted in a moderate increase in rVWF:Ag, reaching approximately 5% of WT levels, even at a 4:1 molar ratio of VWFpp to mutant fl-VWF plasmid (Table 2).

Exogenous supplementation of VWFpp promotes the intracellular processing and trafficking of VWF variants with defective VWFpp

In the present study, the type 2A/IIC VWD variant p.D172N exhibited a significantly reduced intracellular mean fluorescence intensity compared to WT VWF, likely due to increased protein degradation (Figure 3). Furthermore, colocalization of rVWF-D172N with the ER and Golgi apparatus was notably diminished. Co-transfection with WT VWFpp markedly enhanced the intracellular fluorescence intensity of rVWF and facilitated its trafficking from the ER to the Golgi, which was consistent with the significantly increased rVWF:Ag levels in the conditioned medium of AtT-20 cells co-transfected with VWFpp and mutant fl-VWF, compared to cells transfected with mutant fl-VWF alone. Conversely, the three type 3 VWD variants (fl-VWF-S85P, fl-VWF-R273W, and fl-VWF-C295S) showed no

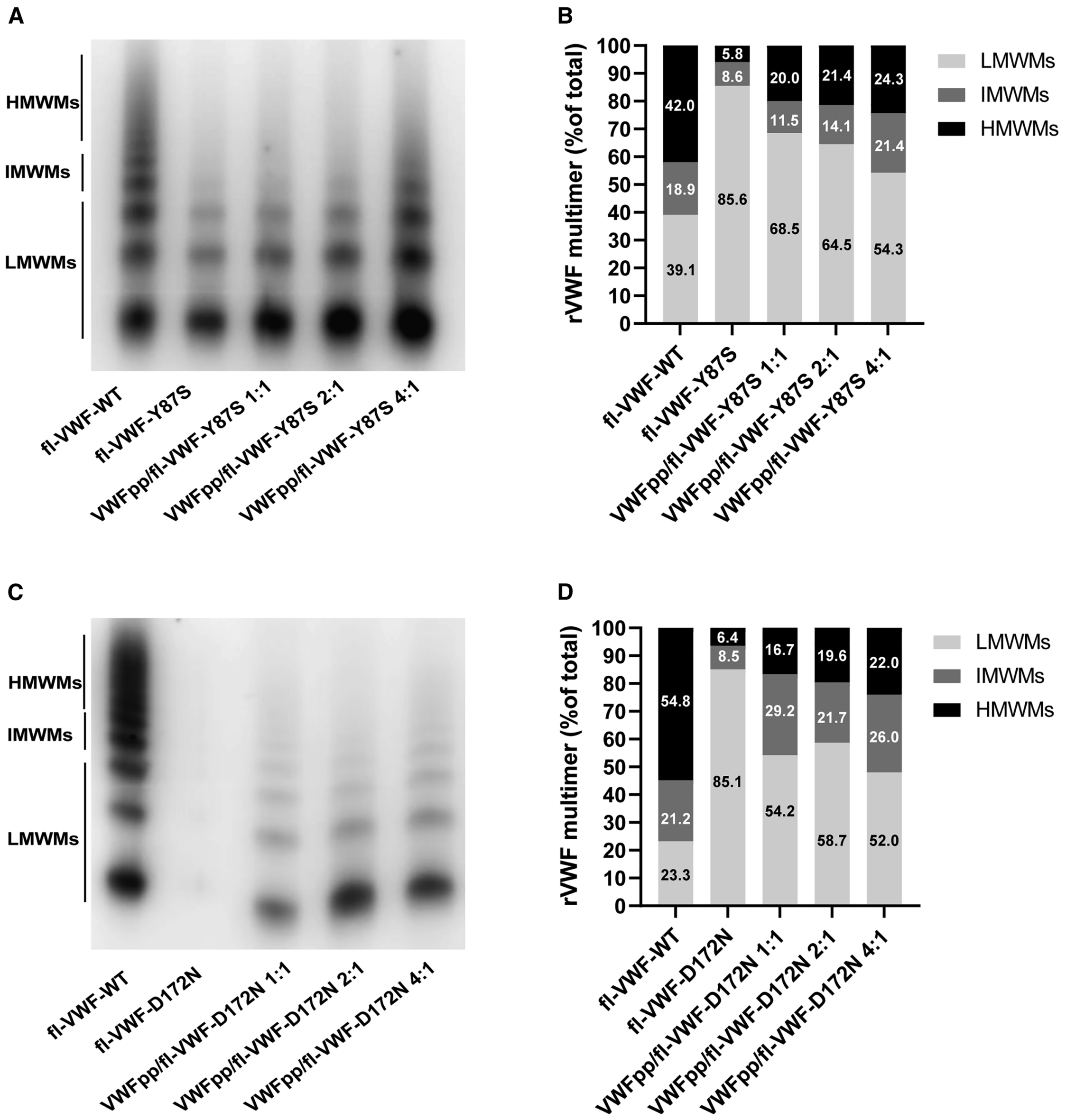

Figure 1. Multimer analysis of recombinant VWF in the conditioned medium of AtT-20 cells co-transfected with wild-type VWFpp and mutant full-length VWF expression plasmids at different molar ratios

(A) Representative image of the VWF multimer pattern in the conditioned medium of AtT-20 cells transiently expressing fl-VWF-Y87S, with or without co-transfection of wild-type (WT) VWFpp. HMWMs, high-molecular-weight VWF multimers (bands >5); IMWMs, intermediate-molecular-weight VWF multimers (bands 4–5); LMWMs, low-molecular-weight VWF multimers (bands 1–3). (B) Relative quantification of HMWMs, IMWMs, and LMWMs from (A), based on band intensity measured using ImageJ software. (C) Representative image of the VWF multimer pattern in the conditioned medium of AtT-20 cells transiently expressing fl-VWF-D172N, with or without co-transfection of WT VWFpp. (D) Relative quantification of HMWMs, IMWMs, and LMWMs from (C), based on band intensity measured using ImageJ software.

significant changes in intracellular fluorescence intensity compared to rVWF-WT. Nonetheless, their colocalization with the ER was significantly increased, suggesting that these mutant proteins were retained within the ER (Figures 3 and S1). Co-transfection with WT VWFpp enhanced the intracellular trafficking of these VWF variants. However, the colocalization of rVWF with both *cis*-Golgi and *trans*-Golgi was still significantly lower compared to rVWF-WT.

Exogenous VWFpp supplementation enhances the tubular storage of type 2A/IIC VWD variants

To characterize the effect of VWFpp co-transfection on the restoration of tubular storage in WPBs, the ultrastructure of rVWF-Y87S and rVWF-D172N before and after exogenous VWFpp supplementation was examined using transmission electron microscopy (Figure 4). WPBs in cells expressing VWF mutants were significantly fewer and

Table 1. *In vitro* restoration of VWF multimerization and secretion by co-expression wild-type VWF propetptide for type 2A VWD variants

VWF variants	Domain	Disrupted steps[a]	Ratio of VWFpp to mutant fl-hVWF plasmid	rVWF:Ag (% of WT)			rVWF: Ag^{lysate}/rVWF: Ag^{medium}		rVWF:CB (% of WT)			rVWF:GP I bM (% of WT)			rVWF:CB/rVWF:Ag			rVWF:GP I bM/rVWF:Ag		
				Pre[b]	Post[c]	Fold increase	Pre[b]	Post[c]	Pre[b]	Post[c]	Fold increase	Pre[b]	Post[c]	Fold increase	Pre[b]	Post[c]	Fold increase	Pre[b]	Post[c]	Fold increase
p.Asp437_Arg442del	D2	1	1:1	66.5 ± 7.7	59.7 ± 6.1	NA	1.0 ± 0.1	0.9 ± 0.3	5.2 ± 3.7	24.6 ± 5.3	4.7	5.8 ± 0.7	30.8 ± 7.9	5.3	0.1 ± 0.1	0.4 ± 0.1	4.0	0.1 ± 0.1	0.5 ± 0.1	5.0
p.Cys570Ser	D2	1	1:1	63.9 ± 4.6	53.3 ± 8.0	NA	0.9 ± 0.1	1.0 ± 0.2	6.7 ± 3.2	19.3 ± 6.9	2.9	5.3 ± 0.7	25.1 ± 2.4	4.7	0.1 ± 0.1	0.4 ± 0.1	4.0	0.1 ± 0.1	0.5 ± 0.1	5.0
p.Ser292_Glu333delinsLys	D1	2	2:1	92.0 ± 10.3	85.3 ± 2.3	NA	0.6 ± 0.1	0.5 ± 0.1	7.2 ± 3.2	33.8 ± 9.2	4.7	13.8 ± 1.5	48.8 ± 1.1	3.5	0.1 ± 0.1	0.4 ± 0.2	4.0	0.1 ± 0.0	0.6 ± 0.0	6.0
p.Cys379Gly	D1	2	2:1	36.9 ± 6.1	66.5 ± 10.3	1.8	0.9 ± 0.2	0.5 ± 0.2	6.7 ± 1.5	34.4 ± 10.0	5.1	6.6 ± 4.5	21.4 ± 4.6	3.2	0.2 ± 0.1	0.5 ± 0.3	2.5	0.2 ± 0.1	0.4 ± 0.1	2.0
p.Gly550Arg	D2	2	4:1	62.8 ± 10.3	82.0 ± 9.9	1.3	0.5 ± 0.1	0.2 ± 0.2	4.6 ± 6.0	38.1 ± 6.0	8.3	10.3 ± 2.8	32.3 ± 3.5	3.1	0.1 ± 0.1	0.5 ± 0.2	5.0	0.2 ± 0.1	0.4 ± 0.0	2.0
p.Tyr87Ser	D1	3	4:1	94.0 ± 2.6	93.9 ± 3.3	1.0	0.7 ± 0.2	0.5 ± 0.2	6.1 ± 0.4	35.2 ± 8.5	5.8	15.8 ± 1.4	62.2 ± 5.1	3.9	0.1 ± 0.0	0.4 ± 0.2	4.0	0.2 ± 0.1	0.7 ± 0.1	3.5
p.Asp172Asn	D1	3	4:1	11.5 ± 6.3	56.9 ± 10.9	5.0	1.0 ± 0.0	0.3 ± 0.0	2.1 ± 1.8	31.4 ± 5.3	15.0	3.9 ± 1.5	42.4 ± 5.5	10.9	0.1 ± 0.2	0.7 ± 0.1	7.0	0.2 ± 0.1	0.7 ± 0.1	3.5
p.Arg202Trp	D1	3	4:1	47.5 ± 10.1	85.7 ± 6.0	1.8	0.9 ± 0.1	0.4 ± 0.1	3.2 ± 1.1	34.4 ± 6.0	10.8	7.4 ± 3.5	63.1 ± 6.2	8.5	0.1 ± 0.1	0.5 ± 0.2	5.0	0.2 ± 0.1	0.8 ± 0.1	4.0

NA, not applicable.

[a]The stepwise assembly of VWF multimer guided by VWFpp: step 1, formation of VWFpp dimers through D2:D2 interactions; step 2, recruitment of the D′D3 domain; step 3, helical stacking of intertwined D1D2D′D3 dimers via D1:D2 interactions.

[b]The levels of rVWF:Ag, rVWF:CB, rVWF:GP I bM , the rVWF:CB/rVWF:Ag ratios, and rVWF:GP I bM/rVWF:Ag ratios in the conditioned medium transfected with mutant fl-hVWF plasmids alone.

[c]The levels of rVWF:GP I bM, the rVWF:CB/rVWF:Ag ratios and rVWF:GP I bM/rVWF:Ag ratios observed after co-transfection of mutant fl-hVWF with hVWFpp plasmids at the indicated molar ratio.

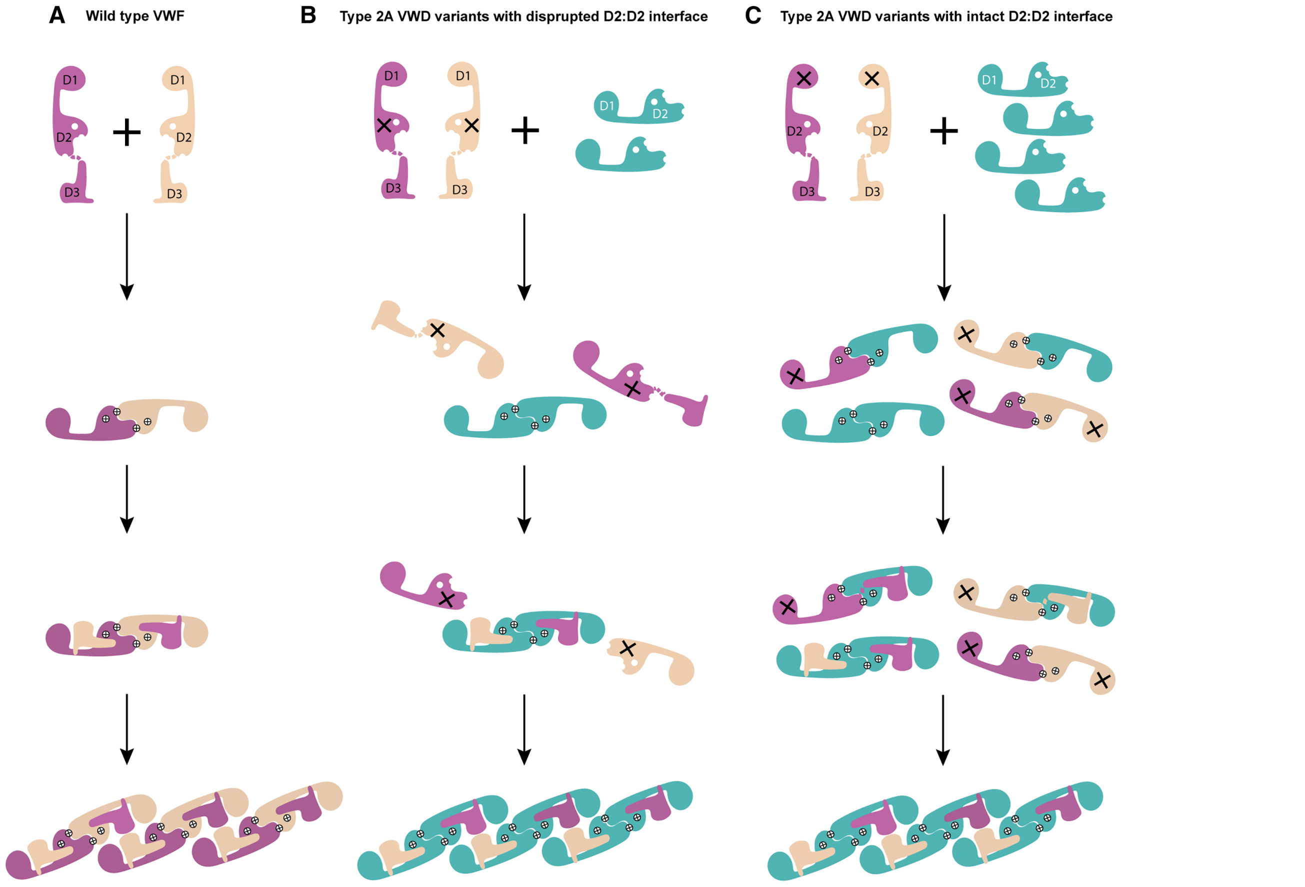

Figure 2. A gene therapy strategy for type 2A VWD through exogenous administration of WT VWF propeptide to restore VWF multimerization

(A) The template mechanism of D1D2 in VWF multimerization. (B) For VWF mutants with defects in the D2:D2 interface, such as VWF-D437_442del, multimerization can be restored by externally supplied WT VWF propeptide (VWFpp). (C) Type 2A VWF mutants with an intact D2:D2 interface, such as VWF-Y87S, retain the ability to form closed proVWF dimers. In these cases, a larger amount of exogenous VWFpp is required to block the closed dimer and subsequently promote multimerization of the mutant VWF.

shorter compared to those in cells expressing rVWF-WT. Moreover, WPBs in mutant VWF-expressing cells exhibited lower electron density, fewer internal striations, and more irregular morphology than those in WT VWF-expressing cells. Following co-transfection with WT VWFpp, although the length of WPBs remained unchanged, their number increased significantly, indicating partial restoration of WPBs formation by co-transfection of VWFpp.

AAV-mediated delivery of mVWFpp restored VWF multimerization and corrects the bleeding phenotype in a type 2A/IIC VWD mouse model

Consistent with the clinical type 2A/IIC VWD phenotype observed in patients, homozygous p.Tyr87Ser mice exhibited normal VWF:Ag levels (68.9% ± 8.5%) and reduced VWF:CB (15.8% ± 10.2%) and VWF:GPIbM (15.0% ± 7.4%), with significantly decreased

Table 2. ***In vitro*** **restoration of VWF secretion by co-expression wild-type VWF propeptide for type 3 VWD variants**

		rVWF:Ag (% of WT)		$rVWF:Ag^{lysate}/rVWF:Ag^{medium}$		rVWF:CB (% of WT)		rVWF:GP I bM (% of WT)		rVWF:CB/rVWF:Ag		rVWF:GP I bM/rVWF:Ag	
VWF variants	Domain	Pre[a]	Post[b]	Pre[a]	Post[b]	Pre[a]	Post[b]	Pre[a]	Post[b]	Pre[a]	Post[b]	Pre[a]	Post[b]
p.Ser85Pro	D1	0.0 ± 0.0	3.4 ± 2.3	NA	11.1 ± 6.7	0.0 ± 0.0	0.5 ± 0.7	0.0 ± 0.0	0.2 ± 0.0	0.0 ± 0.0	0.1 ± 0.1	0.0 ± 0.0	0.1 ± 0.1
p.Arg273Trp	D1	0.0 ± 0.0	4.9 ± 1.6	NA	7.2 ± 3.2	0.0 ± 0.0	0.2 ± 0.1	0.0 ± 0.0	0.2 ± 0.1	0.0 ± 0.0	0.1 ± 0.1	0.0 ± 0.0	0.1 ± 0.1
p.Cys295Ser	D1	1.3 ± 0.1	2.7 ± 0.8	27.6 ± 4.4	10.1 ± 3.7	0.0 ± 0.0	0.4 ± 0.2	0.0 ± 0.0	0.3 ± 0.1	0.0 ± 0.0	0.1 ± 0.1	0.0 ± 0.0	0.1 ± 0.1

NA, not applicable.
[a]The levels of rVWF:Ag, rVWF:CB, rVWF:GP I bM, the rVWF:CB/rVWF:Ag ratios and rVWF:GP I bM/rVWF:Ag ratios in the conditioned medium transfected with mutant full length VWF plasmids alone.
[b]The levels of rVWF:Ag, rVWF:CB, rVWF:GP I bM, the rVWF:CB/rVWF:Ag ratios and rVWF:GP I bM/rVWF:Ag ratios observed after co-transfection of mutant full length VWF with human VWFpp plasmids at a 4:1 molar ratio.

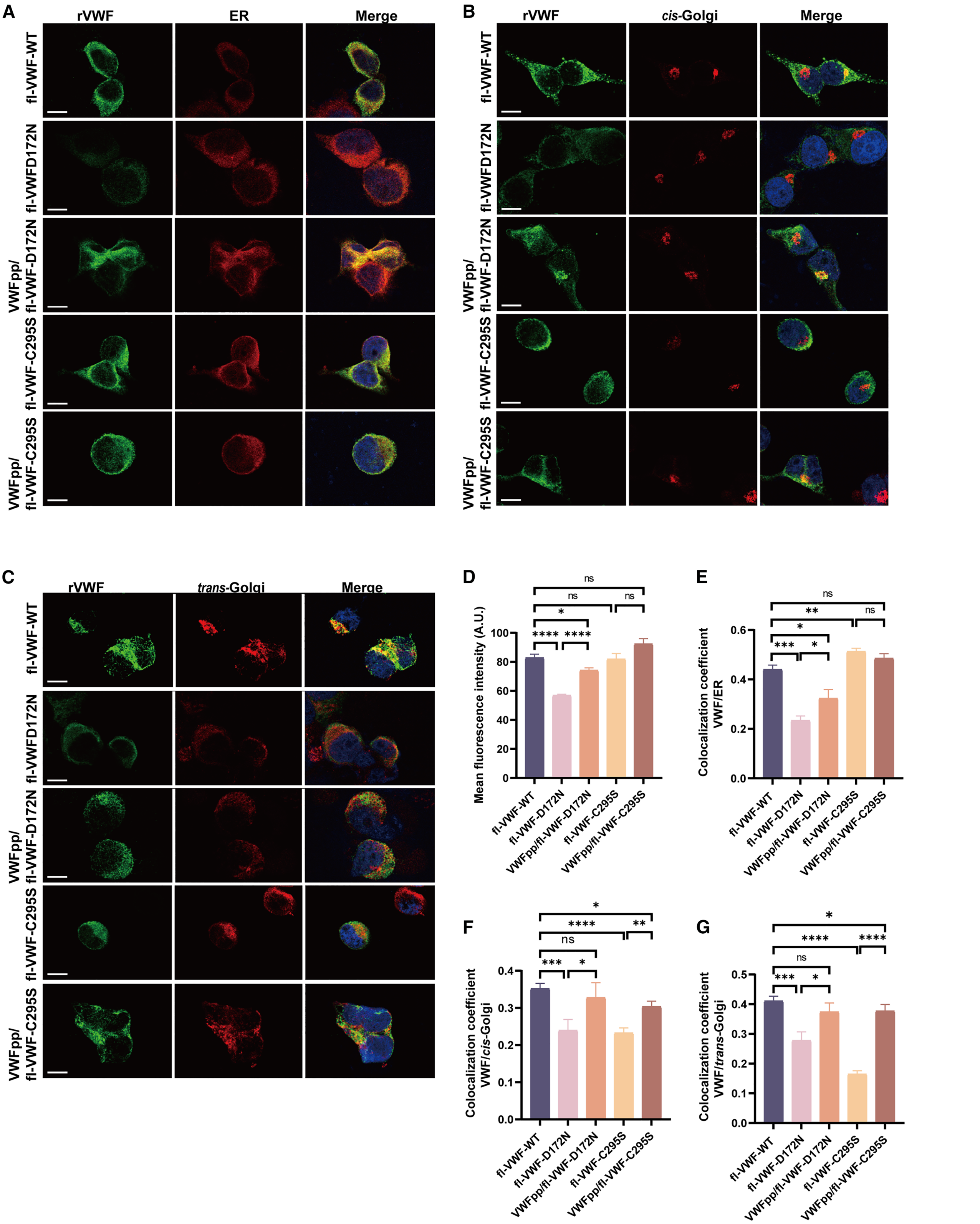

(legend on next page)

FVIII activity levels (6.5% ± 3.0%), a selective loss of HMWMs in plasma, and a pronounced bleeding tendency; heterozygotes showed a mild decrease in VWF:CB (Table S2). VWF:CB levels and HMWMs formation were significantly enhanced 4 weeks post-vector administration (Figures 5A–5H). Administration of the AAV vector driven by the endothelial-specific fms-like receptor tyrosine kinase 1 (FLT1) promoter ensured delivery of mVWFpp to the murine endothelial cells (Figure S2) and resulted in a superior therapeutic effect compared to intercellular adhesion molecule 2 (ICAM2) after 8 weeks, with VWF:Ag levels increasing to 84.5% ± 14.4%; VWF:CB and VWF:GPIbM levels rising to 66.9% ± 21.2% and 47.1% ± 10.4%, respectively; the VWF:CB/VWF:Ag and VWF:GPIbM/VWF:Ag ratios improving to 0.8 ± 0.2 and 0.6 ± 0.1 respectively; and FVIII activity levels increasing to 64.7% ± 37.4% (Figure S3). These therapeutic effects plateaued at 8 weeks and remained stable throughout the 16-week observation period (Figures 5A–5F). Complete blood count analysis of the treated mice revealed no significant abnormalities (Table S3).

The restoration of VWF hemostatic function in type 2A/IIC VWD mice was assessed further using a tail-bleeding model and a $FeCl_3$-induced thrombosis model. Following AAV administration, bleeding times were reduced by approximately 2-fold, and total blood loss volumes were reduced by approximately 4-fold compared to control AAV vector-treated mice (Figures 5I and 5J). Similarly, the time to occlusion in AAV-treated VWD mice was significantly shortened (approximately 3-fold) relative to the control group, reaching levels comparable to those of C57BL/6 WT mice (Figure 5K), demonstrating effective restoration of VWF functional activity.

DISCUSSION

The substantial size of VWF cDNA (8.4 kb) exceeds the packaging limit of conventional AAV vectors,[6] necessitating the exploration of alternative gene therapy approaches for VWD. Structural analyses and *in vitro* expression assays have demonstrated that VWF multimerization is not strictly dependent on VWFpp in *cis*, as exogenously supplied VWFpp in *trans* can effectively facilitate multimer assembly,[12] providing a novel therapeutic strategy targeting VWF variants with impaired VWFpp function.

In this study, we systematically evaluated the capacity of exogenously supplemented VWFpp to restore VWF multimerization and secretion in AtT-20 cells, focusing on eight type 2A/IIC VWD-associated VWF variants and three type 3 VWD-associated variants. For all eight type 2A/IIC VWD variants analyzed, co-transfection with VWFpp increased rVWF:Ag levels to over 50% of WT in the conditioned medium, regardless of baseline rVWF:Ag levels. Moreover, the formation of HMWMs was significantly enhanced, with rVWF:CB and rVWF:GPIbM levels in the conditioned medium increasing from less than 10% to 20%–60% of WT. In general, these findings indicate that exogenously supplemented WT VWFpp can effectively compensate for defective endogenous VWFpp, promoting multimer assembly and the secretion of mature VWF in type 2A/IIC VWD variants.

In contrast, for the three type 3 VWD variants examined, only a modest increase in rVWF:Ag and rVWF:CB levels was observed in the conditioned medium, rising from 0.0% to approximately 5.0% of WT, even at a 4:1 molar ratio of VWFpp to mutant fl-VWF plasmid. Although WT VWFpp may function as an auxiliary chaperone to counteract ER-associated degradation activation caused by misfolded VWF, thereby stabilizing monomeric VWF conformations compatible with Golgi export,[16] its corrective effect on intracellular trafficking of type 3 VWF variants is limited when provided endogenously. The differential corrective effects observed between type 2A/IIC and type 3 variants may be attributed to the intracellular site at which exogenously provided VWFpp exerts its function. In type 2A/IIC VWD variants, VWF trafficking from the ER to the Golgi is largely unaffected or only mildly impaired. When defective endogenous VWFpp is cleaved from mature VWF by furin in the Golgi, exogenously introduced WT VWFpp can effectively compete with the mutant VWFpp, providing a functional template for VWF multimerization and partially restoring multimer formation. In contrast, in type 3 VWD variants, mutations in VWFpp often lead to protein misfolding within the ER, resulting in ER retention and subsequent degradation via the protein quality control mechanisms of the secretory pathway.[17] Most mutant VWF might be degraded before exogenous WT VWFpp can exert its corrective effect.

Notably, exogenously supplemented VWFpp enhanced the VWF multimer profile of type 2A/IIC variants in a dose-dependent manner. The maximal rescue of multimerization was achieved at distinct molar ratios of VWFpp to mutant fl-VWF plasmid, varying among different variants. Variants with defects in the D2:D2 interface and impaired VWFpp dimer formation (e.g., p.Asp437_Arg442-del, p.Cys570Ser) required lower levels of VWFpp for effective multimerization rescue, with maximal correction observed at a 1:1 molar ratio of VWFpp to mutant fl-VWF. In contrast, variants associated with defects in D′D3 recruitment (e.g., p.Ser292_Glu333delinsLys, p.Cys379Gly, p.Gly550Arg) or disruptions in the D1:D2 interface and helical stacking (e.g., p.Tyr87Ser, p.Asp172Asn, p.Arg202Trp) retained the ability to form defective VWFpp dimers. Consequently,

Figure 3. Co-expression of WT VWFpp enhances the intracellular processing and trafficking of VWF variants with defective VWFpp
(A–C) Representative images of AtT-20 cells transiently transfected with WT full-length VWF (fl-VWF-WT), a type 2A VWD variant expression vector (fl-VWF-D172N), or a type 3 VWD variant expression vector (fl-VWF-C295S), or co-transfected with VWFpp and fl-VWF at a 2:1 molar ratio, were stained for VWF (green), the endoplasmic reticulum (ER) (red), the *cis*-Golgi (red), and the *trans*-Golgi (red). Nuclei were stained using 4′,6-diamidino-2-phenylindole (DAPI) (blue). Scale bar, 10 μm. (D) The mean fluorescence intensity of intracellular rVWF-WT, rVWF-D172N, and rVWF-C295S mutants, with and without co-transfection of WT VWFpp, was measured in arbitrary units (A.U.) for at least 50 positively transfected cells from 2 independent experiments. (E–G) The degree of co-localization of different species of rVWF with the ER, *cis*-Golgi, and *trans*-Golgi. Error bars represent the standard error of the mean (SEM). *$p < 0.05$; **$p < 0.01$; ***$p < 0.001$; ****$p < 0.0001$; ns, not statistically significant.

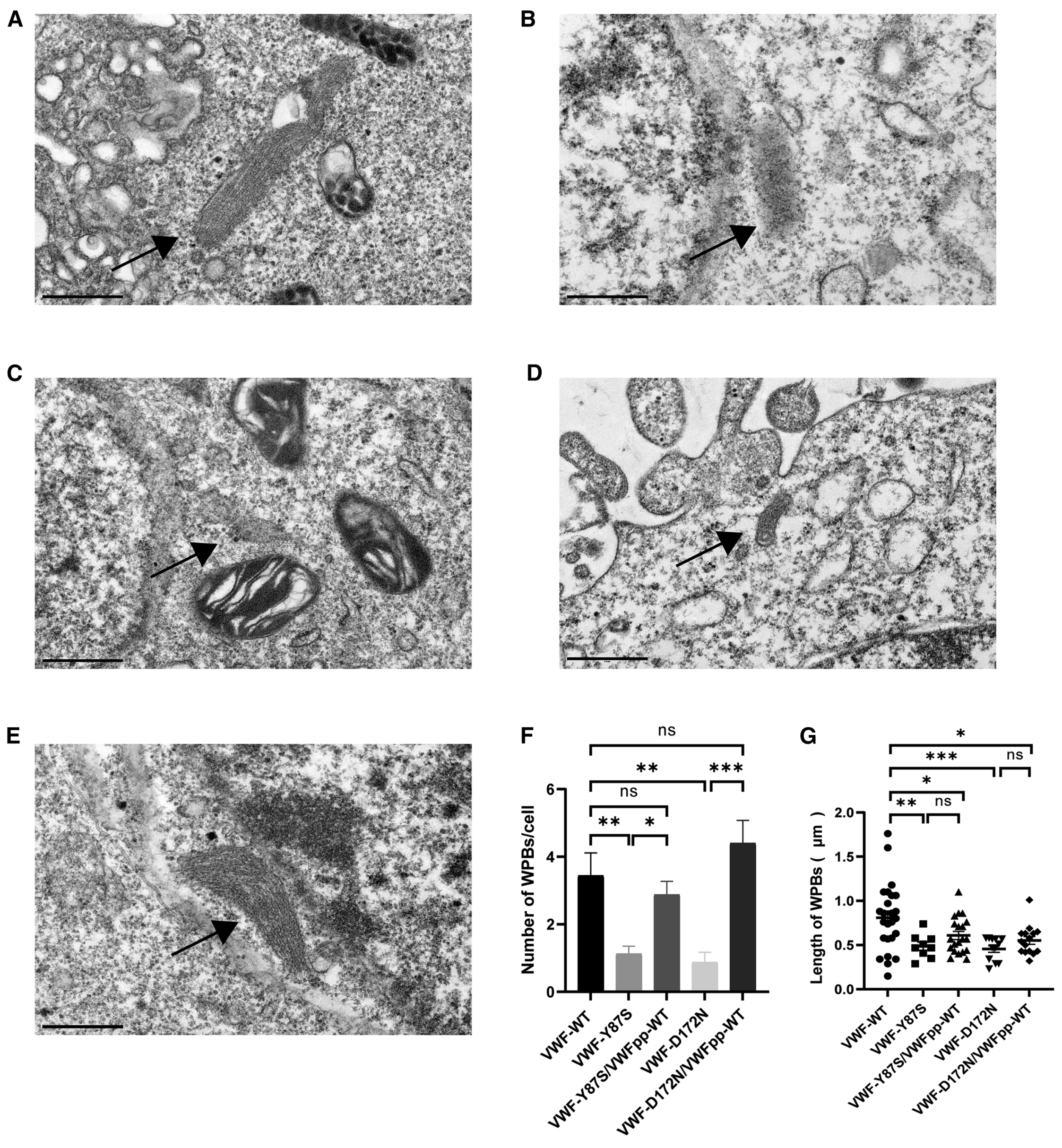

Figure 4. Weibel-Palade body formation in AtT-20 cells following transfection with WT VWF fl-VWF and mutant fl-VWF, as well as co-transfection with VWFpp and mutant fl-VWF

Representative images of AtT-20 cells transiently transfected with either fl-VWF-WT (A), fl-VWF-Y87S (B), or fl-VWF-D172N (C), as well as co-transfected with VWFpp and fl-VWF-Y87S (D) or VWFpp and fl-VWF-D172N (E), using transmission electron microscopy. Scale bar, 500 nm. The number of Weibel-Palade bodies (WPBs) per cell (F) and the length of WPBs (G) were investigated on five different cells from two independent experiments. Data are presented as mean ± SEM in (F), and as median ± interquartile range in (G). *$p < 0.05$; **$p < 0.01$; ***$p < 0.001$; ns, not statistically significant.

these variants exhibited a higher demand for WT VWFpp supplementation, requiring increased molar ratios to achieve optimal VWF multimerization rescue (Figure 2).

Since the cDNA encoding VWFpp is only 2.3 kb in length, well within the packaging capacity of an AAV vector, we explored an experimental gene therapy approach using AAV-mediated delivery of VWFpp in a transgenic mouse model of type 2A/IIC VWD carrying the VWF p.Tyr87Ser mutation. The cDNA encoding WT mVWFpp was inserted into an AAV9 vector, a serotype known for its preferential tropism for endothelial cells and reduced immunogenicity compared to other AAV serotypes.[18,19] To achieve endothelium-restricted expression of VWFpp, its expression was placed under the control of either the FLT1 or ICAM2, both of which have

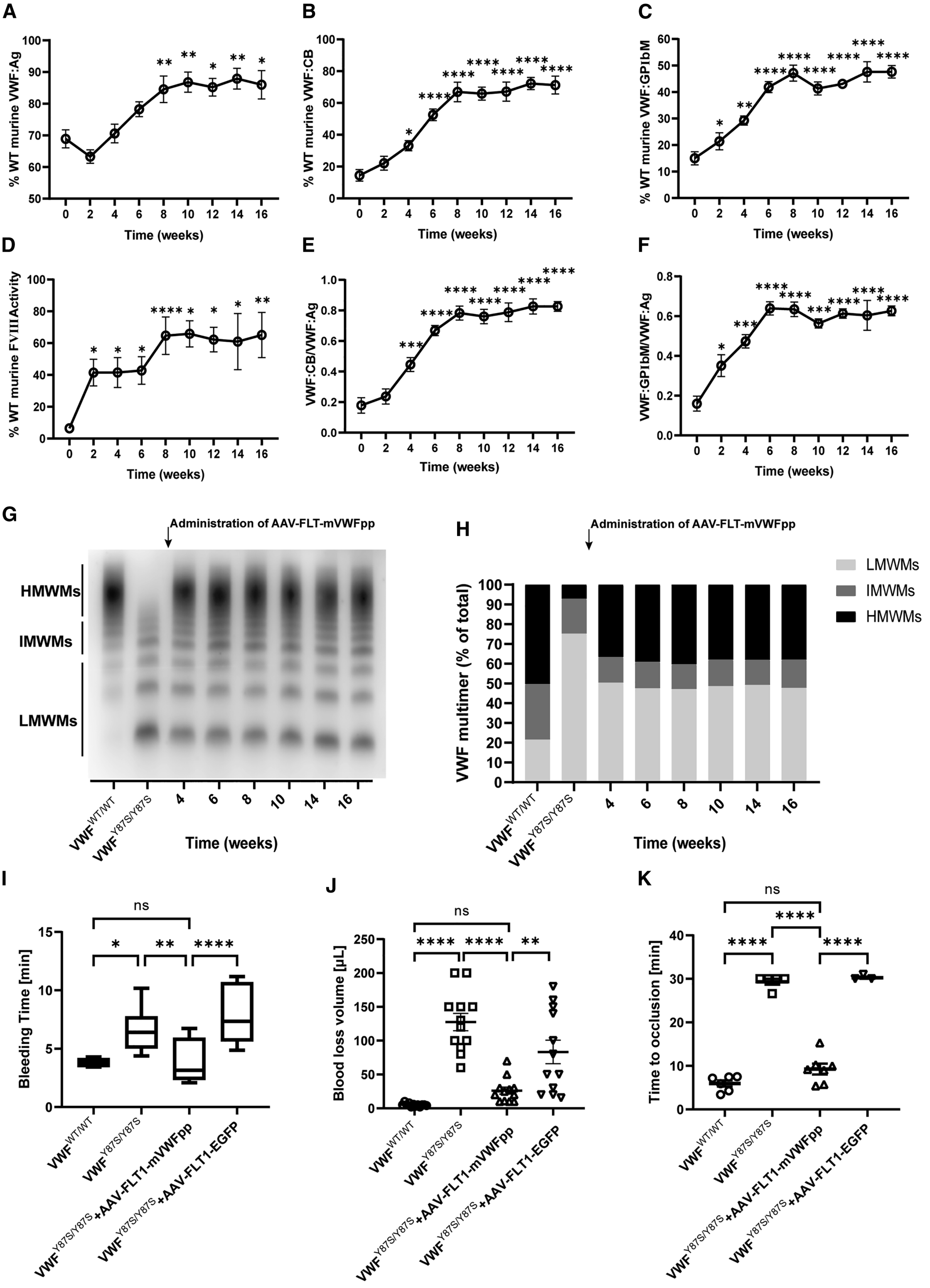

(legend on next page)

demonstrated robust endothelial transduction efficiency.[10,20–22] Notably, the FLT1 promoter exhibited superior therapeutic efficacy compared to ICAM2, likely due to its stronger transcriptional activity in vascular endothelial growth factor (VEGF)-responsive endothelial niches.[23] Tracking and localization studies of AAV9-delivered VWFpp confirmed that the FLT1 promoter facilitated endothelial-specific transduction of murine VWFpp,[24,25] leading to physiologically relevant expression levels in target cells (Figure S2).

At a dosage comparable to that used in gene therapy regimens for hemophilia B,[26] AAV9-mediated VWFpp delivery successfully restored VWF multimer assembly in the type 2A/IIC VWD mouse model, thereby enhancing VWF hemostatic activity and improving the bleeding phenotype. This therapeutic effect was evidenced by significant improvements in both the tail bleeding model and the $FeCl_3$-induced thrombosis model. VWF collagen binding activity increased from 15.8% ± 10.2% before treatment to a peak level of 66.9% ± 21.2% at 8 weeks post-gene delivery and remained stable until the end of the observation period (71.2% ± 12.7% at 16 weeks).

Although the exact mechanism underlying the difference in correction efficiency between the *in vitro* and *in vivo* studies remains unclear, we believe that the superior correction of VWF multimerization observed in the *in vivo* study may be partially attributed to cell type–dependent differences in VWF multimer assembly efficiency. AtT-20 cells, which exhibit higher multimer assembly efficiency, showed greater correction of VWF multimerization for the p.Y87S variant compared to HEK293 and CHO cells upon co-transfection with VWFpp (Figure S4; Table S4). Therefore, it is not surprising that endothelial cells, the physiological site of VWF synthesis and the most competent in forming VWF multimers, achieved the most effective correction in the *in vivo* setting.

Importantly, the treatment was well tolerated, with no major adverse events observed in the treated mice. The findings from this preclinical study highlight the promising potential of this gene therapy strategy for translation into clinical applications for human type 2A/IIC VWD. Future optimization of AAV vectors with enhanced endothelial tropism could further improve therapeutic outcomes by increasing target cell specificity and transduction efficiency. Also, the novel gene therapy strategy by externally delivering VWFpp needs to be investigated in a type 3 VWD model caused by variants in VWFpp in a future study.

In conclusion, the distinctive chaperone function of VWFpp in VWF multimerization allows exogenously supplied VWFpp to compensate for defective endogenous counterparts resulting from VWFpp variants, thereby restoring multimer formation and intracellular transport in type 2A/IIC VWD. AAV-mediated VWFpp gene delivery presents a promising novel therapeutic approach for VWD by addressing the underlying defect at the protein level.

MATERIALS AND METHODS

In vitro cell culture and transient transfection assays

The expression vector for humanfl-VWF-WT was constructed as previously described.[27] Eight type 2A/IIC VWD-associated VWF mutations (p.Tyr87Ser, p.Asp172Asn, p.Arg202Trp, p.Ser292_Glu333delinsLys, p.Cys379Gly, p.Asp437_Arg442del, p.Gly550Arg, and p.Cys570Ser) and three type 3 VWD-associated VWF mutations (p.Ser85Pro, p.Arg273Trp, and p.Cys295Ser) were introduced into the expression vector via site-directed mutagenesis using the KOD-Plus Mutagenesis Kit (TOYOBO, Osaka, Japan). The coding sequence for human VWFpp (residues Met1-Arg763) was amplified from the fl-VWF-WT expression vector and cloned into the pCI-neo plasmid (Promega, Madison, WI) to generate expression construct for VWFpp.

Mouse pituitary tumor cells (AtT-20/D16v-F2, CRL 1795; American Type Culture Collection, Manassas, VA) were cultured and transfected with WT and mutant human fl-VWF expression vectors as previously described.[27] For the rescue assay, VWFpp was co-transfected with mutant fl-VWF expression plasmids at molar ratios of 1:1, 2:1, and 4:1, while maintaining a constant amount of fl-VWF plasmid, to evaluate the impact of *trans*-supplied VWFpp on the restoration of multimerization in VWF mutants. Eight hours post-transfection, the culture medium was refreshed with DMEM (Gibco, Grand Island, NY) supplemented with 1× insulin-transferrin-selenium (Gibco). The conditioned medium was subsequently collected after 36 h. The rVWF:Ag, rVWF:CB, rVWF:GPIbM, and multimer profile of rVWF in the conditioned medium were analyzed according to previously established protocols.[27–29] Three independent experiments were conducted, and the mean values were expressed as a percentage relative to fl-VWF-WT.

Figure 5. Restoration of VWF multimerization and correction of the bleeding phenotype in a type 2A VWD mouse model following AAV-mediated delivery of WT murine VWFpp

The mouse model representing human type 2A VWD caused by the p.Tyr87Ser mutation was administered 5.0×10^{11} vg of an AAV vector delivering WT murine VWFpp via tail vein injection (n = 12). (A–F) VWF antigen levels (VWF:Ag), VWF collagen-binding activity levels (VWF:CB), VWF GPIb-binding activity levels (VWF:GPIbM), FVIII activity levels, and the ratios of VWF:CB/VWF:Ag and VWF:GP I bM/VWF:Ag in mouse plasma before and after AAV administration. (G) Representative image of the VWF multimer pattern in mouse plasma before and after AAV administration. (H) Relative quantification of HMWMs, IMWMs, and LMWMs from (G), based on band intensity measured using ImageJ software. (I) Tail-clipping bleeding times in WT mice (VWFWT/WT), type 2A VWD mice (VWFY87S/Y87S), and type 2A VWD mice 8 weeks after administration of test AAV vectors (AAV-FLT1-mVWFpp) and control AAV vectors (AAV-FLT1-EGFP) (n = 12). (J) Tail-clipping blood loss volume in WT mice (VWFWT/WT), type 2A VWD mice (VWFY87S/Y87S), and type 2A VWD mice 8 weeks after gene transfer (n = 12). (K) Time to occlusion in an FeCl3-induced thrombosis model in WT mice (VWFWT/WT), type 2A VWD mice (VWFY87S/Y87S), and type 2A VWD mice 8 weeks after gene transfer (n = 5). Data are presented as mean ± SEM. *$p < 0.05$; **$p < 0.01$; ****$p < 0.0001$; ns, not statistically significant.

Confocal immunofluorescence microscopy

The intracellular localization of rVWF was assessed by immunofluorescence staining and visualized using a Leica TCS SP8 confocal imaging system, as described previously.[27] Analysis was performed in AtT-20 cells transfected with the type 2A/IIC VWD variant fl-VWF-D172N, which exhibits significantly reduced rVWF:Ag levels, and three type 3 VWD variants (fl-VWF-S85P, fl-VWF-R273W, and fl-VWF-C295S), which have impaired secretion, with or without co-transfection of VWFpp. Primary antibodies used included a rabbit polyclonal anti-human VWF antibody (5 μg/mL) (Dako, Santa Clara, CA), a mouse monoclonal anti-Serca2 ATPase antibody (5 μg/mL) (Invitrogen, Carlsbad, CA) for ER marking, a mouse monoclonal anti-GM130 antibody (5 μg/mL) (BD Biosciences, San Jose, CA) for *cis*-Golgi marking, and a mouse monoclonal immunoglobulin G1κ TGN38 antibody (5 μg/mL) (Santa Cruz Biotechnology, Dallas, TX) for *trans*-Golgi marking. Analysis of each comparison set was performed on a minimum of 50 positively transfected cells from 2 independent experiments. The mean fluorescence intensity of intracellular rVWF was quantified using ImageJ software, and the degree of colocalization was calculated using the mean Pearson's correlation coefficient.

Transmission electron microscopy

The number and morphology of WPBs exceeding 350 nm in length were assessed by transmission electron microscopy in cells transfected with WT or mutant type 2A/IIC VWD variants (fl-VWF-Y87S and fl-VWF-D172N), as well as those co-transfected with VWFpp and mutant fl-VWF.[30] For each comparison set, five distinct cells from two independent experiments were analyzed.

Viral vector construction

The AAV expression vector for mVWFpp was generated by inserting the mVWFpp coding sequence into an AAV plasmid under the control of the endothelia-specific promoter FLT1 or ICAM2. The control plasmid pAAV-EGFP contained cDNA encoding EGFP as a reporter. Figure S5 presents the AAV vector plasmid maps for both control and test vectors. Recombinant AAV9 vectors were generated via a triple-transfection method as previously described,[31] using control or test AAV plasmids, an adenoviral helper plasmid, and a plasmid encoding the AAV2 rep gene and the AAV9 cap gene. Viral particles were purified by iodixanol gradient ultracentrifugation, and vector titers (viral genomes [vg]/mL) were quantified by quantitative real-time polymerase chain reaction.

Generation of type 2A/IIC VWD mouse models

The type 2A/IIC VWD mouse model with a known human type 2A/IIC VWF variant p.Tyr87Ser on the C57BL/6J background was generated by CRISPR-Cas9 strategy.[32] The guide RNA (gRNA) targeting exon 4 of the mouse *Vwf* gene, along with a donor oligonucleotide containing the p.Tyr87Ser variant and a synonymous p.Val86Val mutation, and Cas9 were co-injected into fertilized mouse zygotes to create targeted knockin offspring. The p.Val86Val synonymous mutation was included to prevent gRNA recognition and recutting of the sequence after homology-directed repair. The resulting founder mice were crossed with C57BL/6J mice to establish type 2A/IIC VWD lineages. Heterozygous $VWF^{Y87S/WT}$ offspring were then bred to generate the homozygous $VWF^{Y87S/Y87S}$ model as well as WT $VWF^{WT/WT}$ control mice.

Transduction specificity

Mouse lung tissue was fixed in paraformaldehyde, paraffin embedded, sectioned, and rehydrated. Sections underwent heat-induced epitope retrieval in EDTA buffer (pH 9.0) under pressure, followed by blocking with 10% goat serum for 30 min and overnight incubation at 4°C with anti-GFP primary antibody (Abcam, Cambridge, UK; 1:200 dilution). Nuclei were counterstained with 4′,6-diamidino-2-phenylindole (DAPI) (Solarbio, Beijing, China; 1:500 dilution). Fluorescence images were acquired using an Olympus microscope (Tokyo, Japan), and fluorescence intensity was quantified using ImageJ software.

In vivo studies

All animal procedures followed protocols approved by the Institutional Animal Care and Use Committee of Ruijin Hospital (TACU22-0080). Twelve adult male type 2A/IIC mice (6–8 weeks old) were intravenously injected with 5.0×10^{11} vg/mouse of both test and control AAV vectors. Whole blood was collected into buffered citrate by phlebotomy of the retro-orbital plexus at different times points post-vector administration. Plasma was obtained by centrifuging for 3 min at 13,000 rpm. Analysis of VWF parameters (VWF:Ag, VWF:CB, VWF:GPIbM, FVIII activity, and multimer profile) in mouse plasma was performed before and after vector administration, using the aforementioned methods. Results are shown as mean ± standard error of the mean (SEM).

The *in vivo* hemostatic efficacy of mVWFpp delivery was assessed by measuring tail clipping bleeding times and occlusion times using a ferric chloride-induced thrombosis model 8 weeks after vector administration.[33,34] In the tail-clip model, the tails of mice were severed at a 2.5-mm diameter and monitored for bleeding or clotting for 30 min, with bleeding time and total amount of blood loss recorded. Twelve mice per group were analyzed. For the ferric chloride-induced thrombosis model, mice were anesthetized with 1% pentobarbital sodium, and the common carotid arteries were exposed. Vascular injury was induced by applying a filter paper saturated with 10% $FeCl_3$ to the left carotid artery for 3 min. A transit-time perivascular flow probe (Model 0.5 PSB, Transonic Systems, Ithaca, NY) was placed on the carotid artery surface to monitor the time to thrombus formation and occlusion. If occlusion time exceeded 30 min, then the experiment was terminated. Five mice per group were analyzed.

DATA AVAILABILITY

All data and materials are shown in the paper and/or the supplemental information. Further inquiries can be directed to the corresponding authors.

ACKNOWLEDGMENTS

This study was supported by the General Program of the National Natural Science Foundation of China (grant nos. 82070137, 82170128, 82270128, and 82270129) and a grant from the National Key R&D Program of China (2023YFC2507800).

AUTHOR CONTRIBUTIONS

Z.Z.: data curation, methodology, investigation, formal analysis, and writing – original draft. Q.L.: data curation, methodology, project administration, and writing – review & editing. X.X.: methodology, investigation, and formal analysis. Y.L.: methodology. C.C.: investigation. Q.D.: supervision and funding acquisition. A.Z.: supervision and data curation. W.W.: writing – review & editing, supervision, and conceptualization. X.W.: supervision, project administration, funding acquisition, and conceptualization. J.D.: writing – review & editing, supervision, project administration, funding acquisition, and conceptualization.

DECLARATION OF INTERESTS

The authors declare no competing interests.

SUPPLEMENTAL INFORMATION

Supplemental information can be found online at https://doi.org/10.1016/j.ymthe.2025.09.027.

REFERENCES

1. Leebeek, F.W.G., and Eikenboom, J.C.J. (2016). Von Willebrand's Disease. N. Engl. J. Med. *375*, 2067–2080.
2. Connell, N.T., Flood, V.H., Brignardello-Petersen, R., Abdul-Kadir, R., Arapshian, A., Couper, S., Grow, J.M., Kouides, P., Laffan, M., Lavin, M., et al. (2021). ASH ISTH NHF WFH 2021 guidelines on the management of von Willebrand disease. Blood Adv. *5*, 301–325.
3. Federici, A.B. (2008). The use of desmopressin in von Willebrand disease: the experience of the first 30 years (1977-2007). Haemophilia *14*, 5–14.
4. Ben-Ami, T., and Revel-Vilk, S. (2013). The use of DDAVP in children with bleeding disorders. Pediatr. Blood Cancer *60*, S41–S43.
5. Mannucci, P.M., Chediak, J., Hanna, W., Byrnes, J., Ledford, M., Ewenstein, B.M., Retzios, A.D., Kapelan, B.A., Schwartz, R.S., and Kessler, C.; Alphanate Study Group (2002). Treatment of von Willebrand disease with a high-purity factor VIII/von Willebrand factor concentrate: a prospective, multicenter study. Blood *99*, 450–456.
6. De Meyer, S.F., Vanhoorelbeke, K., Chuah, M.K., Pareyn, I., Gillijns, V., Hebbel, R. P., Collen, D., Deckmyn, H., and VandenDriessche, T. (2006). Phenotypic correction of von Willebrand disease type 3 blood-derived endothelial cells with lentiviral vectors expressing von Willebrand factor. Blood *107*, 4728–4736.
7. Wang, L., Rosenberg, J.B., De, B.P., Ferris, B., Wang, R., Rivella, S., Kaminsky, S.M., and Crystal, R.G. (2012). In vivo gene transfer strategies to achieve partial correction of von Willebrand disease. Hum. Gene Ther. *23*, 576–588.
8. Barbon, E., Kawecki, C., Marmier, S., Sakkal, A., Collaud, F., Charles, S., Ronzitti, G., Casari, C., Christophe, O.D., Denis, C.V., et al. (2023). Development of a dual hybrid AAV vector for endothelial-targeted expression of von Willebrand factor. Gene Ther. *30*, 245–254.
9. Yee, A., Gildersleeve, R.D., Gu, S., Kretz, C.A., McGee, B.M., Carr, K.M., Pipe, S.W., and Ginsburg, D. (2014). A von Willebrand factor fragment containing the D'D3 domains is sufficient to stabilize coagulation factor VIII in mice. Blood *124*, 445–452.
10. Campioni, M., Legendre, P., Loubiere, C., Lunghi, B., Pinotti, M., Christophe, O.D., Lenting, P.J., Denis, C.V., Bernardi, F., and Casari, C. (2021). In vivo modulation of a dominant-negative variant in mouse models of von Willebrand disease type 2A. J. Thromb. Haemost. *19*, 139–146.
11. Yadegari, H., Halimeh, S., Krahforst, A., Pavlova, A., Pezeshkpoor, B., Müller, J., Pötzsch, B., Biswas, A., Marquardt, N., Scholz, U., et al. (2025). Landscape and spectrum of VWF variants in type 2 Von Willebrand Disease: insights from a German patient cohort. Thromb. Haemost. *5*. https://doi.org/10.1055/a-2616-5161.
12. Zeng, J., Shu, Z., Liang, Q., Zhang, J., Wu, W., Wang, X., and Zhou, A. (2022). Structural basis of von Willebrand factor multimerization and tubular storage. Blood *139*, 3314–3324.
13. Schneppenheim, R., Budde, U., and Ruggeri, Z.M. (2001). A molecular approach to the classification of von Willebrand disease. Best Pract. Res. Clin. Haematol. *14*, 281–298.
14. Wise, R.J., Pittman, D.D., Handin, R.I., Kaufman, R.J., and Orkin, S.H. (1988). The propeptide of von Willebrand factor independently mediates the assembly of von Willebrand multimers. Cell *52*, 229–236.
15. Haberichter, S.L., Allmann, A.M., Jozwiak, M.A., Montgomery, R.R., and Gill, J.C. (2009). Genetic alteration of the D2 domain abolishes von Willebrand factor multimerization and trafficking into storage. J. Thromb. Haemost. *7*, 641–650.
16. Javed, A., Christodoulou, J., Cabrita, L.D., and Orlova, E.V. (2017). The ribosome and its role in protein folding: looking through a magnifying glass. Acta Crystallogr. D Struct. Biol. *73*, 509–521.
17. Araki, K., and Nagata, K. (2011). Protein folding and quality control in the ER. Cold Spring Harb. Perspect. Biol. *3*, a007526.
18. Varadi, K., Michelfelder, S., Korff, T., Hecker, M., Trepel, M., Katus, H.A., Kleinschmidt, J.A., and Müller, O.J. (2012). Novel random peptide libraries displayed on AAV serotype 9 for selection of endothelial cell-directed gene transfer vectors. Gene Ther. *19*, 800–809.
19. Bozoglu, T., Lee, S., Ziegler, T., Jurisch, V., Maas, S., Baehr, A., Hinkel, R., Hoenig, A., Hariharan, A., Kim, C.I., et al. (2022). Endothelial Retargeting of AAV9 In Vivo. Adv. Sci. *9*, e2103867.
20. Portier, I., Vanhoorelbeke, K., Verhenne, S., Pareyn, I., Vandeputte, N., Deckmyn, H., Goldenberg, D.S., Samal, H.B., Singh, M., Ivics, Z., et al. (2018). High and long-term von Willebrand factor expression after sleeping beauty transposon-mediated gene therapy in a mouse model of severe von Willebrand disease. J. Thromb. Haemost. *16*, 592–604.
21. de Jong, A., Dirven, R.J., Boender, J., Atiq, F., Anvar, S.Y., Leebeek, F.W.G., van Vlijmen, B.J.M., and Eikenboom, J. (2020). Ex vivo Improvement of a von Willebrand Disease Type 2A Phenotype Using an Allele-Specific Small-Interfering RNA. Thromb. Haemost. *120*, 1569–1579.
22. Jongejan, Y.K., Schrader Echeverri, E., Dirven, R.J., Paunovska, K., Linthorst, N.A., de Jong, A., Wellershoff, J.C., van der Gouw, K.D., van Vlijmen, B.J.M., Dahlman, J. E., and Eikenboom, J.C.J. (2023). Small interfering RNA-mediated allele-selective silencing of von Willebrand factor in vitro and in vivo. Blood Adv. *7*, 6108–6119.
23. Verma, M., Asakura, Y., Wang, X., Zhou, K., Ünverdi, M., Kann, A.P., Krauss, R.S., and Asakura, A. (2024). Endothelial cell signature in muscle stem cells validated by VEGFA-FLT1-AKT1 axis promoting survival of muscle stem cell. Elife *13*, e73592.
24. Antoniou, K.M., Soufla, G., Proklou, A., Margaritopoulos, G., Choulaki, C., Lymbouridou, R., Samara, K.D., Spandidos, D.A., and Siafakas, N.M. (2009). Different activity of the biological axis VEGF-Flt-1 (fms-like tyrosine kinase 1) and CXC chemokines between pulmonary sarcoidosis and idiopathic pulmonary fibrosis: a bronchoalveolar lavage study. Clin. Dev. Immunol. *2009*, 537929.
25. Volpe, M.C., Ciucci, G., Zandomenego, G., Vuerich, R., Ring, N.A.R., Vodret, S., Salton, F., Marchesan, P., Braga, L., Marcuzzo, T., et al. (2023). Flt1 produced by lung endothelial cells impairs ATII cell transdifferentiation and repair in pulmonary fibrosis. Cell Death Dis. *14*, 437.
26. Ishikawa, K., Weber, T., and Hajjar, R.J. (2018). Human Cardiac Gene Therapy. Circ. Res. *123*, 601–613.
27. Liang, Q., Zhang, Z., Ding, B., Shao, Y., Ding, Q., Dai, J., Hu, X., Wu, W., and Wang, X. (2024). A noncanonical splicing variant c.875-5 T > G in von Willebrand factor causes in-frame exon skipping and type 2A von Willebrand disease. Thromb. Res. *236*, 51–60.
28. Vanhoorelbeke, K., Cauwenberghs, N., Vauterin, S., Schlammadinger, A., Mazurier, C., and Deckmyn, H. (2000). A reliable and reproducible ELISA method to measure ristocetin cofactor activity of von Willebrand factor. Thromb. Haemost. *83*, 107–113.
29. Yin, J., Ma, Z., Su, J., Wang, J.W., Zhao, X., Ling, J., Bai, X., Ouyang, W., Wang, Z., Yu, Z., and Ruan, C. (2015). Mutations in the D1 domain of von Willebrand factor impair their propeptide-dependent multimerization, intracellular trafficking and secretion. J. Hematol. Oncol. *8*, 73.
30. You, L., Wang, X., Wu, W., Nepovimova, E., Wu, Q., and Kuca, K. (2022). HIF-1α inhibits T-2 toxin-mediated "immune evasion" process by negatively regulating PD-1/PD-L1. Toxicology *480*, 153324.

31. Manno, C.S., Pierce, G.F., Arruda, V.R., Glader, B., Ragni, M., Rasko, J.J., Ozelo, M. C., Hoots, K., Blatt, P., Konkle, B., et al. (2006). Successful transduction of liver in hemophilia by AAV-Factor IX and limitations imposed by the host immune response. Nat. Med. *12*, 342–347.

32. Rosenberg, J.B., Haberichter, S.L., Jozwiak, M.A., Vokac, E.A., Kroner, P.A., Fahs, S.A., Kawai, Y., and Montgomery, R.R. (2002). The role of the D1 domain of the von Willebrand factor propeptide in multimerization of VWF. Blood *100*, 1699–1706.

33. Nair, N., De Wolf, D., Nguyen, P.A., Pham, Q.H., Samara-Kuko, E., Landau, J., Blouse, G.E., Chuah, M.K., and VandenDriessche, T. (2021). Gene therapy for hemophilia B using CB 2679d-GT: a novel factor IX variant with higher potency than factor IX Padua. Blood *137*, 2902–2906.

34. De Meyer, S.F., Vandeputte, N., Pareyn, I., Petrus, I., Lenting, P.J., Chuah, M.K.L., VandenDriessche, T., Deckmyn, H., and Vanhoorelbeke, K. (2008). Restoration of plasma von Willebrand factor deficiency is sufficient to correct thrombus formation after gene therapy for severe von Willebrand disease. Arterioscler. Thromb. Vasc. Biol. *28*, 1621–1626.

YMTHE, Volume *33*

Supplemental Information

Correction of VWF multimerization in type 2A/IIC von Willebrand disease by exogenous VWF propeptide supplementation

Ziqi Zhang, Qian Liang, Xiaoqian Xu, Yang Li, Changming Chen, Qiulan Ding, Aiwu Zhou, Wenman Wu, Xuefeng Wang, and Jing Dai

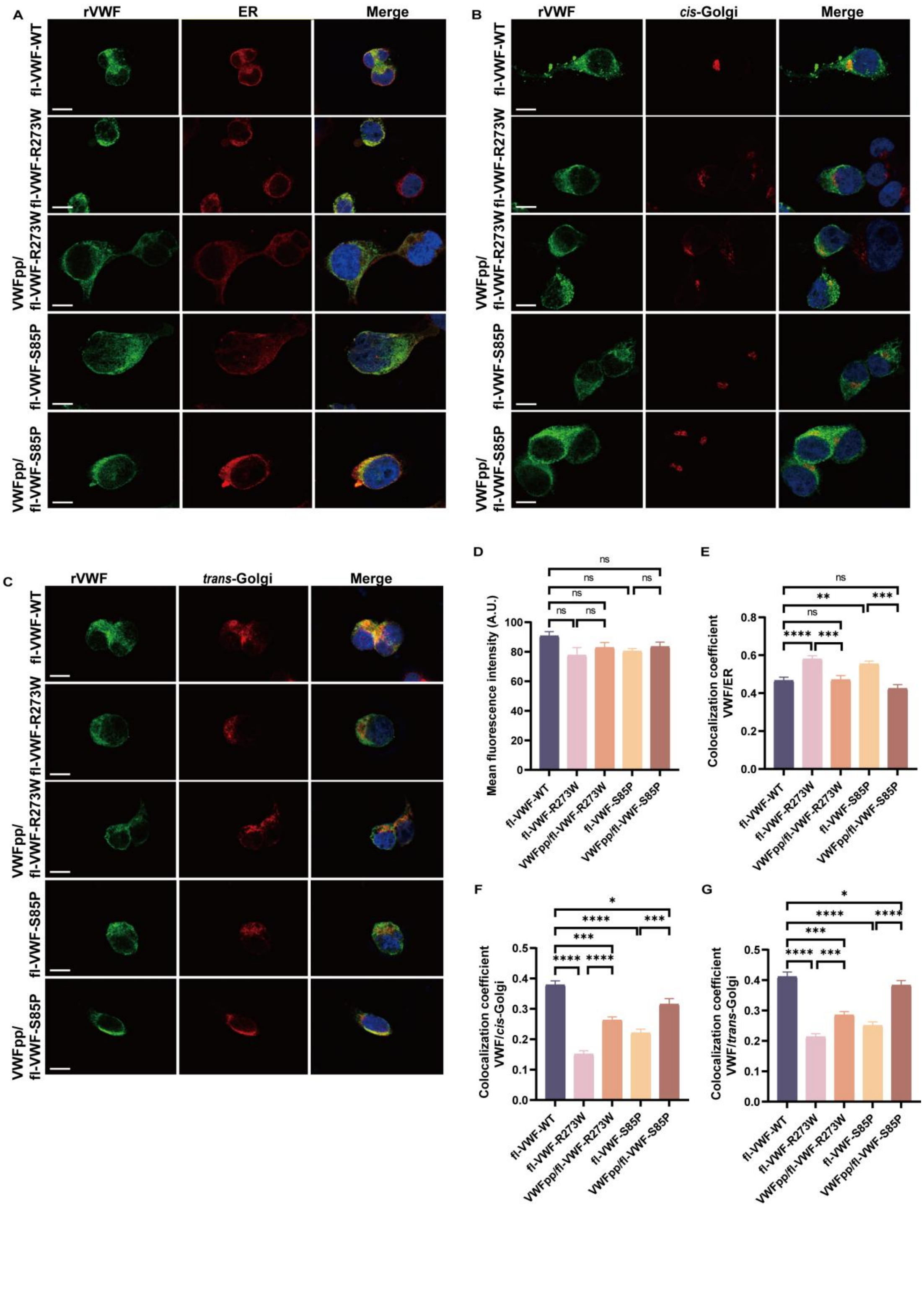

A
rVWF
ER
Merge
fl-VWF-WT
fl-VWF-R273W
VWFpp/ fl-VWF-R273W
fl-VWF-S85P
VWFpp/ fl-VWF-S85P
B
rVWF
cis-Golgi
Merge
C
rVWF
trans-Golgi
Merge
D
Mean fluorescence intensity (A.U.)
E
Colocalization coefficient VWF/ER
F
Colocalization coefficient VWF/cis-Golgi
G
Colocalization coefficient VWF/trans-Golgi
fl-VWF-WT
fl-VWF-R273W
VWFpp/fl-VWF-R273W
fl-VWF-S85P
VWFpp/fl-VWF-S85P
ns

Figure S1. Co-transfection with a plasmid expressing wild-type VWFpp improves intracellular trafficking of the type 3 VWD-causing variants.

(A-C) Representative images of AtT-20 cells transiently transfected with wild-type full-length VWF (fl-VWF-WT) or expression vectors for type 3 VWD-causing variants (fl-VWF-R273W and fl-VWF-S85P), or co-transfected with VWFpp and fl-VWF at a 2:1 molar ratio, were stained for VWF (green), the endoplasmic reticulum (ER) (red), the *cis*-Golgi (red), and the *trans*-Golgi (red). Nuclei were stained with 4’,6-diamidino-2-phenylindole (DAPI, blue). Scale bar = 10 μm. (D) The mean fluorescence intensity of intracellular rVWF-WT, rVWF-R273W, and rVWF-S85P mutants, with and without co-transfection of wild-type VWFpp, was measured in arbitrary units (A.U.) for at least 50 positively transfected cells from two independent experiments. (E-G) The degree of co-localization of different species of rVWF with the ER, *cis*-Golgi and *trans*-Golgi. Error bars represent the standard error of mean (SEM). *, $P < 0.05$; **, $P < 0.01$; ***, $P < 0.001$; ****, $P < 0.0001$; ns, not statistically significant.

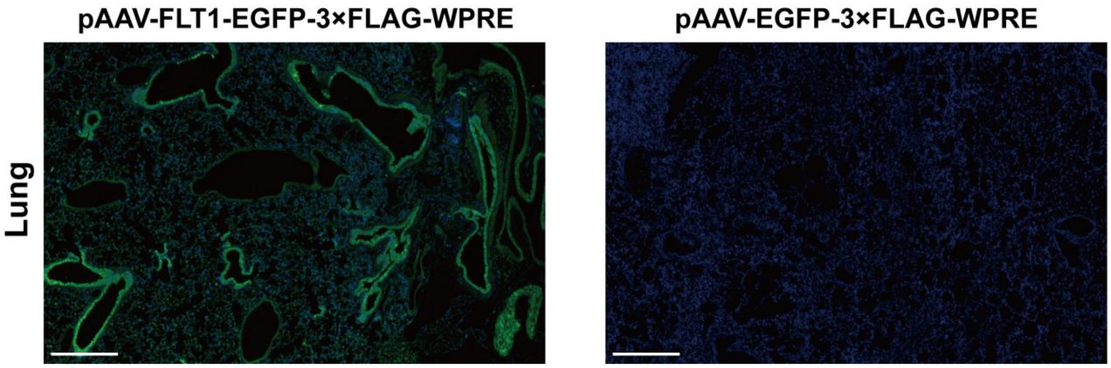

Figure S2. Representative GFP immunofluorescence staining of mouse lung tissue. The left panel shows a mouse injected with the AAV expression vector for mVWFpp under the control of the endothelial-specific promoter fms-like tyrosine kinase 1 (FLT1). The right panel shows a mouse injected with the control AAV vector lacking the FLT1 promoter. Green fluorescence indicates GFP expression, and blue fluorescence indicates DAPI nuclear staining. Scale bar: 500 μm.

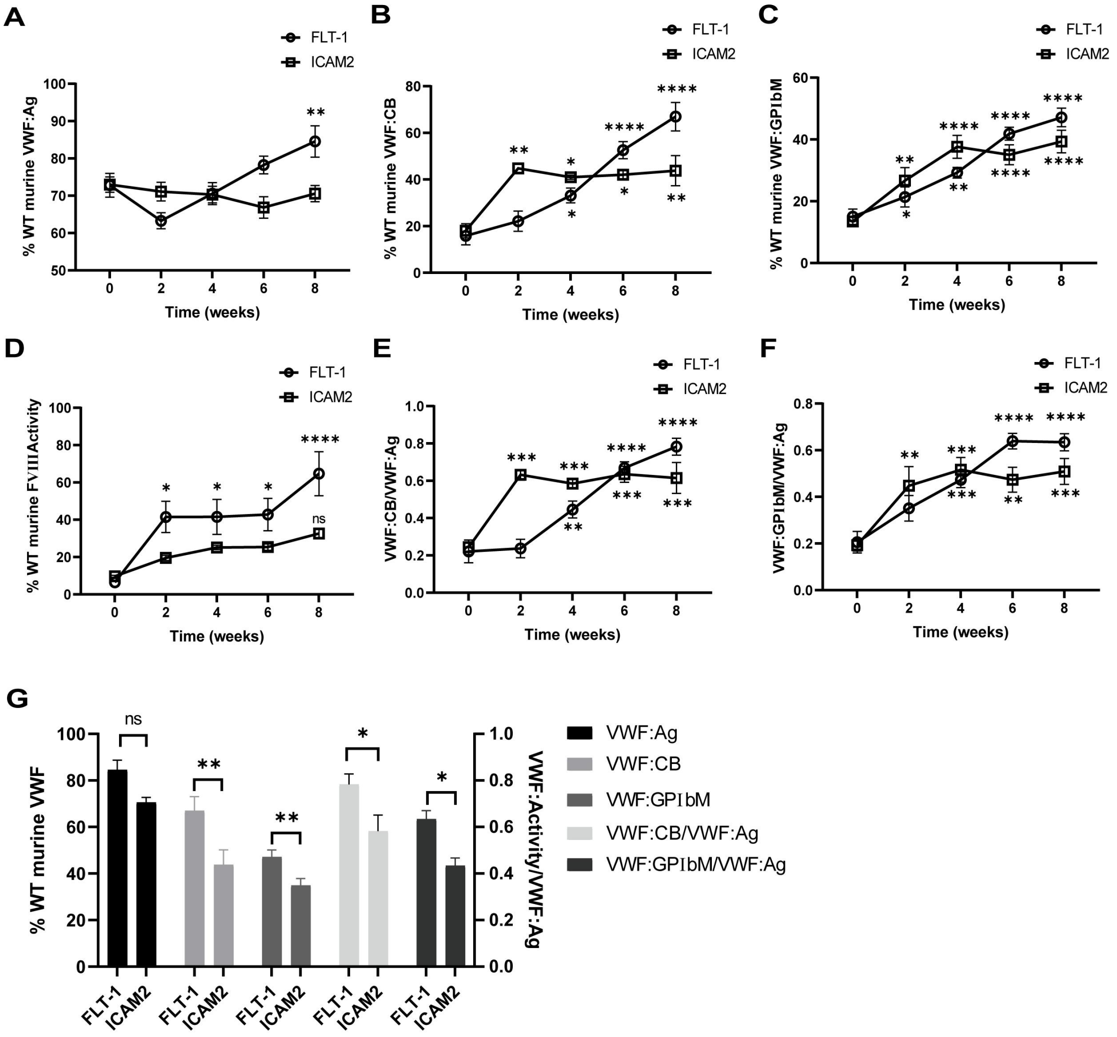

Figure S3. Restoration of VWF activity in a type 2A VWD mouse model via AAV-mediated delivery of wild-type murine VWF propeptide under the control of two distinct endothelial-specific promoters. (A-F) VWF antigen levels (VWF:Ag), VWF collagen binding activity levels (VWF:CB), VWF GPIb-binding activity levels (VWF:GPIbM), FVIII activity levels and the ratios of VWF:CB/VWF:Ag and VWF:GPIbM/VWF:Ag in type 2A VWD mouse plasma (VWF$^{Y87S/Y87S}$) before and after tail vein administration of AAV vectors delivering wild-type VWFpp at a dose of 5.0 × 10^{11} vg per mouse. Gene expression was driven by either the endothelial-specific fms-like receptor tyrosine kinase 1 (FLT1) or intercellular adhesion molecule 2 (ICAM2) promoter (n = 8). (G) Comparison of VWF:Ag levels, VWF:CB levels, VWF:GPIbM levels, VWF:CB/VWF:Ag ratios and VWF:GPIbM/VWF:Ag ratios between the two promoter groups 8 weeks post-gene transfer. Data are presented as mean ± standard error of the mean (SEM). *, P < 0.05; **, P < 0.01; ***, P < 0.001; ****, P < 0.0001; ns, not statistically significant.

A

Cell lines	rVWF:Ag	rVWF:CB	rVWF:GPIbM	rVWF:CB/rVWF:Ag	rVWF:GPIbM/rVWF:Ag
AtT20	100.0 ± 0.0	100.0 ± 0.0	100.0 ± 0.0	1.0 ± 0.0	1.0 ± 0.0
HEK293	83.8 ± 5.7	75.0 ± 6.4	82.4 ± 7.3	0.9 ± 0.0	0.9 ± 0.1
CHO	75.2 ± 7.2	67.5 ±10.1	57.1 ± 6.6	0.9 ± 0.1	0.8 ± 0.0

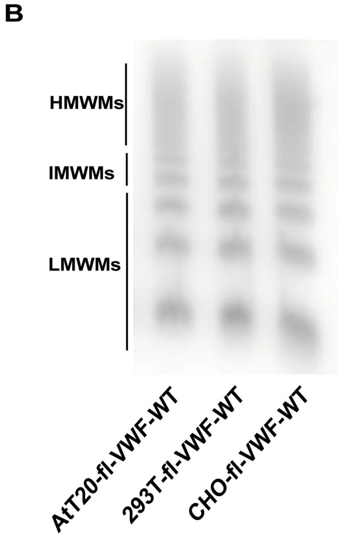

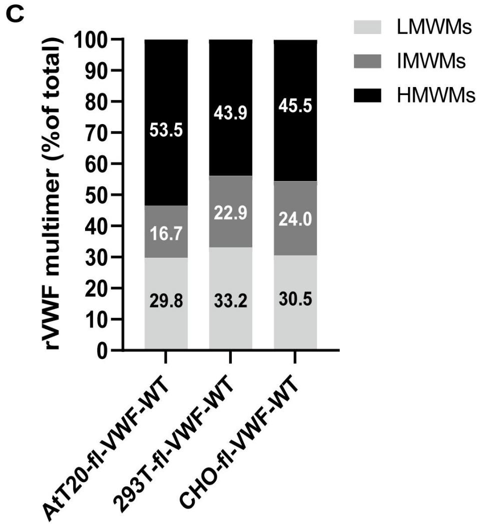

Figure S4. Expression of full-length wild-type recombinant VWF in AtT-20, HEK293, and CHO cells. (A) *In vitro* expression of full-length wild-type recombinant VWF performed in three different cell lines. VWF expression levels in HEK293, and CHO cells were calculated as a percentage of those expressed in AtT20 cells. (B) Representative image of the VWF multimer pattern in the conditioned medium of AtT-20 cells, HEK 293 cells and CHO cells transiently expressing full-length wild-type VWF. HMWMs: high-molecular-weight VWF multimers (bands >5); IMWMs: intermediate-molecular-weight VWF multimers (bands 4-5); LMWMs: low-molecular-weight VWF multimers (bands 1-3). (C) Relative quantification of HMWMs, IMWMs, and LMWMs from panel B, based on band intensity measured using Image J software.

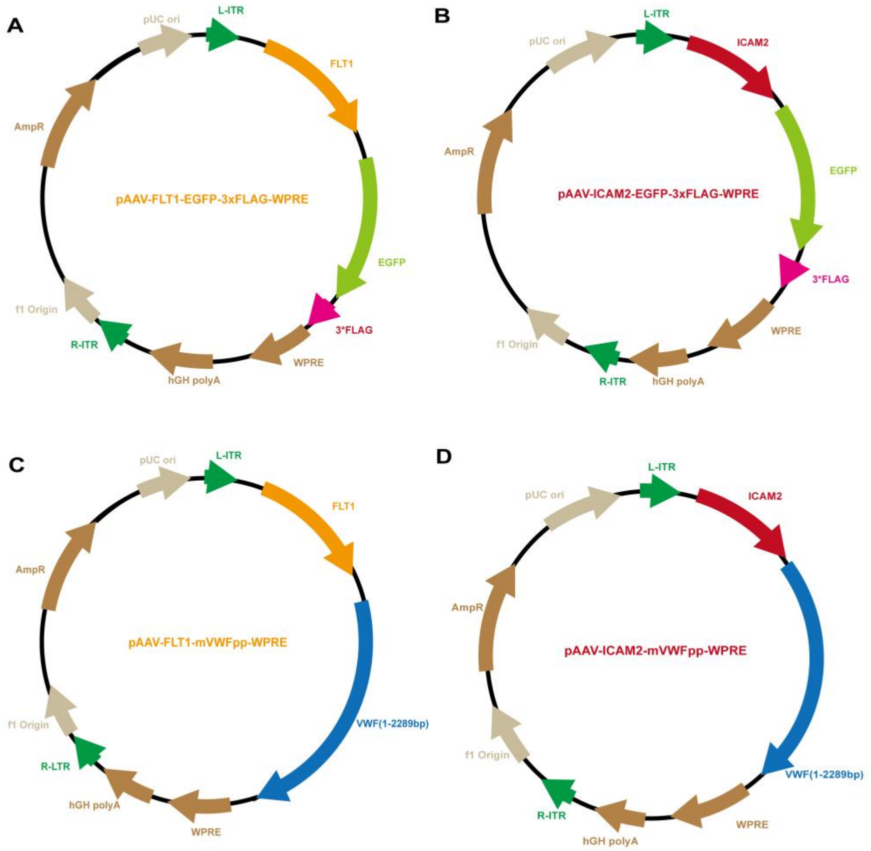

Figure S5. Maps of AAV vector plasmids.
(A-B) Schematic representation of control AAV vector plasmids utilizing fms-like receptor tyrosine kinase 1 (FLT1) or intercellular adhesion molecule 2 (ICAM2) as endothelial-specific promoters. The control vectors also contain additional elements, including enhanced green fluorescent protein (EGFP) as a reporter, a triple FLAG peptide (3×FLAG), and the woodchuck hepatitis virus posttranscriptional regulatory element (WPRE). (C-D) Schematic representation of test AAV vector plasmids expressing murine VWF propeptide (VWFpp), generated by replacing the EGFP and triple FLAG peptide coding regions with the wild-type murine VWFpp sequence.

Table S1. *In vitro* restoration of VWF multimerization and secretion by co-expression of wild-type VWF propeptide with type 2A VWD variants at different molar ratios.

VWFpp, VWF propetide; fl-hVWF, full length human VWF;[a], the stepwise assembly of VWF multimer guided by VWFpp. Step i, formation of VWFpp dimers through D2:D2 interactions; step ii, recruitment of the D'D3 domain; step iii, helical stacking of intertwined D1D2D'D3 dimers via D1:D2 interactions. [b], The levels of rVWF:Ag, rVWF:CB, rVWF:GPIbM, the rVWF:CB/rVWF:Ag ratios and rVWF:GPIbM/rVWF:Ag ratios in the conditioned medium transfected with mutant fl-hVWF plasmids alone. [c], The levels of rVWF:Ag, rVWF:CB, rVWF:GPIbM, the rVWF:CB/rVWF:Ag ratios and rVWF:GPIbM/rVWF:Ag ratios observed after co-transfection of mutant fl-hVWF with hVWFpp plasmids at various molar ratios. The molar ratios of hVWFpp to mutant fl-hVWF plasmids that achieved the best correction effect are highlighted in bold. NA, not applicable.

Table S2. VWF phenotypic characterization in wild-type and type 2A/IIC mice ($VWF^{Y87S/Y87S}$).

Genotype	VWF:Ag	VWF:CB	VWF:CB/VWF:Ag
$VWF^{WT/WT}$(wild-type)	84.8±15.8	80.2±21.8	0.9±0.2
$VWF^{Y87S/Y87S}$(homozygotes)	68.9±8.5	15.8±10.2	0.2±0.1
$VWF^{Y87S/WT}$(heterozygotes)	68.1±10.8	41.9±17.9	0.6±0.2

Table S3. Complete blood count analysis in the type 2A/IIC VWD mouse model (VWF$^{Y87S/Y87S}$) 16 weeks post vector administration.

WBC, White Blood Cell Count (Reference range: 4.45-13.96×10^9/L); NEU(%), Neutrophil Percentage (Reference range: 7.36-28.59%); LYM(%), Lymphocyte Percentage (Reference range: 61.26-87.18%); MON(%), Monocyte Percentage (Reference range: 2.18-11.02%); EOS(%), Eosinophil Percentage (Reference range: 0.13-4.42%); BAS(%), Basophil Percentage (Reference range: 0.01-1.24%); NEU(10^9/L), Neutrophil Count (Reference range: 0.53-3.09×10^9/L); LYM(10^9/L), Lymphocyte Count (Reference range: 3.24-11.15×10^9/L); MON(10^9/L), Monocyte Count (Reference range: 0.15-0.94×10^9/L); EOS(10^9/L), Eosinophil Count (Reference range: 0.01-0.42×10^9/L); BAS(10^9/L), Basophil Count (Reference range: 0-0.13×10^9/L); RBC, Red Blood Cell Count (Reference range: 7.14-12.2×10^{12}/L); HGB, Hemoglobin Concentration (Reference range: 108.0-192.0 g/L); HCT, Hematocrit (Reference range: 37.3-.0%); MCV, Mean Corpuscular Volume (Reference range: 42.7-56.0 fL); MCH, Mean Corpuscular Hemoglobin (Reference range: 11.7-16.3 pg); MCHC, Mean Corpuscular Hemoglobin Concentration (Reference range: 246.0-349.0 g/L); RDW-SD, Red Blood Cell Distribution Width- Standard Deviation (Reference range: 23.0-39.0 fL); RDW-CV, Red Blood Cell Distribution Width-Coefficient of Variation (Reference range: 12.0-19.0%); PL, Platelet Count (Reference range: 841-2159×10^9/L); MPV, Mean Platelet Volume (Reference range: 4.3-6.1 fL); PCT, Platelet Hematocrit (Reference range: 0.1-0.78%); PDW, Platelet Distribution Width (Reference range: 12.0-17.5%).

Table S4. *In vitro* restoration of VWF multimerization by co-expression wild-type VWF propetptide for p.Tyr87Ser variant in three cell lines.

Cell lines	rVWF:Ag (% of WT)		rVWF:CB (% of WT)		rVWF:GPIbM(% of WT)		rVWF:CB/rVWF:Ag		rVWF:GPIbM/rVWF:Ag	
	Pre[a]	Post[b]	Pre[a]	Post[b]	Pre[a]	Post[b]	Pre[a]	Post[b]	Pre[a]	Post[b]
AtT20	111.1 ± 11.8	90.9 ± 11.3	8.2 ± 2.2	37.3 ± 7.3	28.1 ± 6.4	60.6 ± 9.5	0.1 ± 0.0	0.4 ± 0.2	0.2 ± 0.1	0.6 ± 0.1
HEK293	87.1 ± 8.4	69.6 ± 7.0	5.4 ± 4.2	15.4 ± 2.3	21.8 ± 6.3	30.9 ± 8.1	0.1 ± 0.1	0.2 ± 0.1	0.2 ± 0.2	0.4 ± 0.2
CHO	81.4 ± 9.2	57.1 ± 7.7	2.1 ± 1.3	12.0 ± 2.5	26.4 ± 9.1	27.4 ± 9.1	0.1 ± 0.0	0.2 ± 0.1	0.2 ± 0.2	0.4 ± 0.1

[a], The levels of rVWF:Ag, rVWF:CB, and the rVWF:CB/rVWF:Ag ratios in the conditioned medium transfected with mutant full length VWF plasmids alone. [b], The levels of rVWF:Ag, rVWF:CB, and the rVWF:CB/rVWF:Ag ratios observed after co-transfection of mutant full length VWF with human VWFpp plasmids at a 4:1 molar ratio.
